# On-device synaptic memory consolidation using Fowler-Nordheim quantum-tunneling

**DOI:** 10.3389/fnins.2022.1050585

**Published:** 2023-01-13

**Authors:** Mustafizur Rahman, Subhankar Bose, Shantanu Chakrabartty

**Affiliations:** Department of Electrical and Systems Engineering, Washington University in St. Louis, St. Louis, MO, United States

**Keywords:** hardware synapse, memory consolidation, quantum-tunneling, neuromorphic, continual learning

## Abstract

**Introduction:**

For artificial synapses whose strengths are assumed to be bounded and can only be updated with finite precision, achieving optimal memory consolidation using primitives from classical physics leads to synaptic models that are too complex to be scaled *in-silico*. Here we report that a relatively simple differential device that operates using the physics of Fowler-Nordheim (FN) quantum-mechanical tunneling can achieve tunable memory consolidation characteristics with different plasticity-stability trade-offs.

**Methods:**

A prototype FN-synapse array was fabricated in a standard silicon process and was used to verify the optimal memory consolidation characteristics and used for estimating the parameters of an FN-synapse analytical model. The analytical model was then used for large-scale memory consolidation and continual learning experiments.

**Results:**

We show that compared to other physical implementations of synapses for memory consolidation, the operation of the FN-synapse is near-optimal in terms of the synaptic lifetime and the consolidation properties. We also demonstrate that a network comprising FN-synapses outperforms a comparable elastic weight consolidation (EWC) network for some benchmark continual learning tasks.

**Discussions:**

With an energy footprint of femtojoules per synaptic update, we believe that the proposed FN-synapse provides an ultra-energy-efficient approach for implementing both synaptic memory consolidation and continual learning on a physical device.

## 1. Introduction

**Figure 1:**
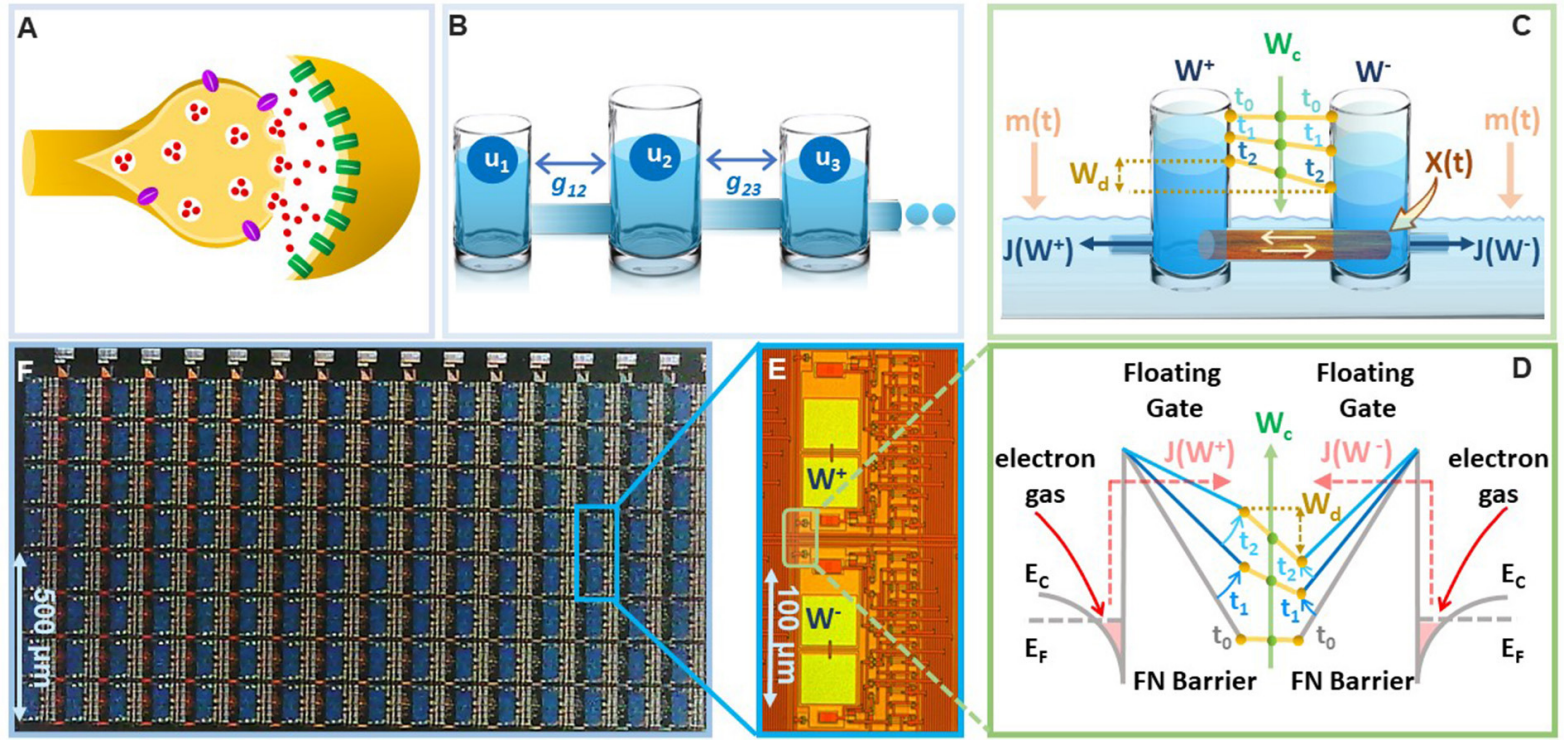
On-device memory consolidation using FN-synapses: **(A)** An illustration of a biological synapse with different coupled biochemical processes that determine synaptic dynamics **(B)** physical realization of the cascade model reported in Benna and Fusi (2016) that captures the consolidation dynamics using fluid in reservoirs *u*_*k*_ that are coupled through parameters *g*_*kj*_. **(C)** Illustration of the FN-synapse dynamics using a differential reservoir model and its state at time-instants *t*_0_, *t*_1_, and *t*_2_; **(D)** energy-band diagram to show the implementation of the reservoir model in **(C)** using the physics of Fowler-Nordheim quantum-mechanical tunneling where a single synaptic element (as show in **E**) which stores the weight *W*_*d*_ as the differential charge stored between each tunneling junction, i.e., $W_{d}=\frac{W^{+}-W^{-}}{2}$ and the common-mode tunneling voltage *W*_*c*_ as the average of the individual charges, i.e., $W_{c}=\frac{W^{+}+W^{-}}{2}$); **(E)** micrograph of a single FN-synapse; **(F)** micrograph of an array of FN-synaptic devices fabricated in a standard silicon process.

There is a growing evidence from the field of neuroscience and neuroscience inspired AI about the importance of implementing synapses as a complex high-dimensional dynamical system (Fusi et al., 2005; Benna and Fusi, 2016), as opposed to a simple and a static storage element, as depicted in standard neural networks (Sohoni et al., 2019). This dynamical systems viewpoint has been motivated by the hypothesis that complex interactions between plethora of biochemical processes at a synapse (illustrated in Figure 1A) produces *synaptic metaplasticity* (Abraham, 2008) and plays a key role in *synaptic memory consolidation* (Li et al., 2017). Both these phenomena have been observed in biological synapses (Yang et al., 2009, 2014) where the synaptic plasticity (or ease of update) can vary depending on age and task-specific usage that is accumulated during the process of learning. In literature these long-term synaptic memory consolidation dynamics have been captured using different analytical models with varying degrees of complexity (Amit and Fusi, 1994; Fusi, 2002; Fusi et al., 2005; Fusi and Abbott, 2007; Roxin and Fusi, 2013; Benna and Fusi, 2016). One such model is the cascade model (Benna and Fusi, 2016) which has been shown to achieve the theoretically optimal memory consolidation characteristic for benchmark random pattern experiments. However, the physical realization of cascade models as described in Benna and Fusi (2016) uses a complex coupling of dynamical states and diffusion dynamics (an example illustrated in Figure 1B using a reservoir model), which is difficult to mimic and scale *in-silico*. Similar optimal memory consolidation characteristics have been reported in the context of continual learning in artificial neural networks (ANN) where synapses that are found to be important for learning a specific task are consolidated (or become rigid) (Aljundi et al., 2017; Kirkpatrick et al., 2017; Lee et al., 2017; Zenke et al., 2017; Chaudhry et al., 2018; Liu et al., 2018). As a result, when learning a new task the synaptic weight does not significantly deviate from the consolidated weights, hence, the network seeks solutions that work well for as many tasks as possible. However, these synaptic models are algorithmic in nature and it is not clear if the optimal consolidation characteristics can be naturally implemented on the synaptic device *in-silico*. Also, it is not clear if the consolidation properties of the physical synaptic device can be tuned to achieve different *plasticity-stability* trade-offs and hence can overcome the relative disadvantages of the EWC models. In this paper, we report that a simple differential device that operates using the physics of Fowler-Nordheim (FN) quantum-mechanical tunneling can achieve tunable synaptic memory consolidation characteristics similar to the algorithmic consolidation models. The operation of the synaptic device, referred to in this paper as the FN-synapse, can be understood using a reservoir model as shown in Figure 1C). Two reservoirs with fluid levels *W*^+^ and *W*^−^ are coupled to each other using a sliding barrier X. The barrier is used to control the fluid flow from the respective reservoirs into an external medium. The respective flows, which are modeled by functions *J*(*W*^+^) and *J*(*W*^−^), at time-instant *t* are modulated by the position of the sliding barrier *X*(*t*) and the level of fluid in the external reservoir *m*(*t*). In this reservoir model, the synaptic weight is stored as $W_{d}=\frac{1}{2}\left(W^{+}-W^{-}\right)$ whereas $W_{c}=\frac{1}{2}\left(W^{+}+W^{-}\right)$ serves as an indicator of synaptic usage with respect to time.

In the Section 3, we show that for a synapse based on a general differential reservoir model [without making assumptions on the nature of the flow function *J*(.)] the synaptic weight *W*_*d*_ evolves in response to the external input *X*(*t*) according to the coupled differential equation


(1) $$\begin{array}{l} \frac{dW_{d}}{dt}=-r\left(t\right)W_{d}+X\left(t\right) \end{array}$$

where


(2) $$\begin{array}{l} r\left(t\right)=\frac{d^{2}W_{c}}{dt^{2}}{\left(\frac{dW_{c}}{dt}\right)}^{-1} \end{array}$$

is a time varying decay function that models the dynamics of the synaptic plasticity as a function of the history of synaptic activity (or its usage). The usage parameter *W*_*c*_ evolves according to


(3) $$\begin{array}{l} \frac{dW_{c}}{dt}=-J\left(W_{c}\right)+m\left(t\right) \end{array}$$

based on the functions *J*(.) and *m*(*t*). Equations (1)–(3) show that the weight *W*_*d*_ update does not directly depend on the non-linear function *J*(.) but implicitly through the common-mode *W*_*c*_. Furthermore, Equation (1) conforms to the weight update equation reported in the EWC model (Kirkpatrick et al., 2017) where it has been shown that if *r*(*t*) varies according to the network Fisher information metric, then the strength of a stored pattern or memory (typically defined in terms of signal-to-noise ratio) decays at an optimal rate of $1/\sqrt{t}$ when the synaptic network is subjected to random, uncorrelated memory patterns. In Section 3, we show that if the objective is to maximize the operational lifetime of the synapse, then equating the time-evolution profile in Equation (2) to r(t)≈O(1/t) (Kirkpatrick et al., 2017) leads to an optimal *J*(.) of the form *J*(*V*) ∝ *V*^2^exp(−β/*V*) where β is a constant. The expression for *J*(*V*) matches the expression for a Fowler-Nordheim (FN) quantum-mechanical tunneling current (Lenzlinger and Snow, 1969) indicating that optimal synaptic memory consolidation could be achieved on a physical device operating on the physics of FN quantum-tunneling.

To verify on-device optimal consolidation dynamics we fabricated an array of FN-synapses and Figures 1D, E show the micrograph of the fabricated prototype. In the Section 3, we show the mapping of the differential reservoir model using the physical variables associated with FN quantum tunneling and Figure 1F shows the mapping using an energy-band diagram. Similar to our previous works (Zhou and Chakrabartty, 2017; Zhou et al., 2019; Rahman et al., 2022), the tunneling junctions have been implemented using polysilicon, silicon-di-oxide, and n-well layers, where the silicon-di-oxide forms the FN-tunneling barrier for electrons to leak out from the n-well onto a polysilicon layer. The polysilicon layer forms a floating-gate where the initial charge can be programmed using a combination of hot-electron injection or quantum-tunneling (Mehta et al., 2020, 2022). The synaptic weight is stored as a differential voltage $W_{d}=\frac{1}{2}\left(W^{+}-W^{-}\right)$ across two floating-gates as shown in Figure 1F. The voltages on the floating-gates *W*^+^ and *W*^−^ at any instant of time are modified by the differential signals $\pm \frac{1}{2}X\left(t\right)$ that are coupled onto the floating-gates. The dynamics for updating *W*^+^ and *W*^−^ are determined by the respective tunneling currents *J*(.) which discharge the floating-gates. In the Supplementary Figure 1, we describe the complete equivalent circuit for the FN-synapse along with the read-out mechanism used in this work to measure *W*_*d*_. The presence of additional coupling capacitors in Supplementary Figure 1 provides a mechanism to inject a common-mode modulation signal *m*(*t*) into the FN-synapse. We will show in the Section 2 that *m*(*t*) can be used to tune the memory consolidation characteristics of the FN-synapse array to achieve memory capacity similar to or better than the cascade consolidation models (with different degrees of complexities) or the task-specific synaptic consolidation corresponding to the EWC model.

## 2. Results

### 2.1. FN-synapse characterization

**Figure 2:**
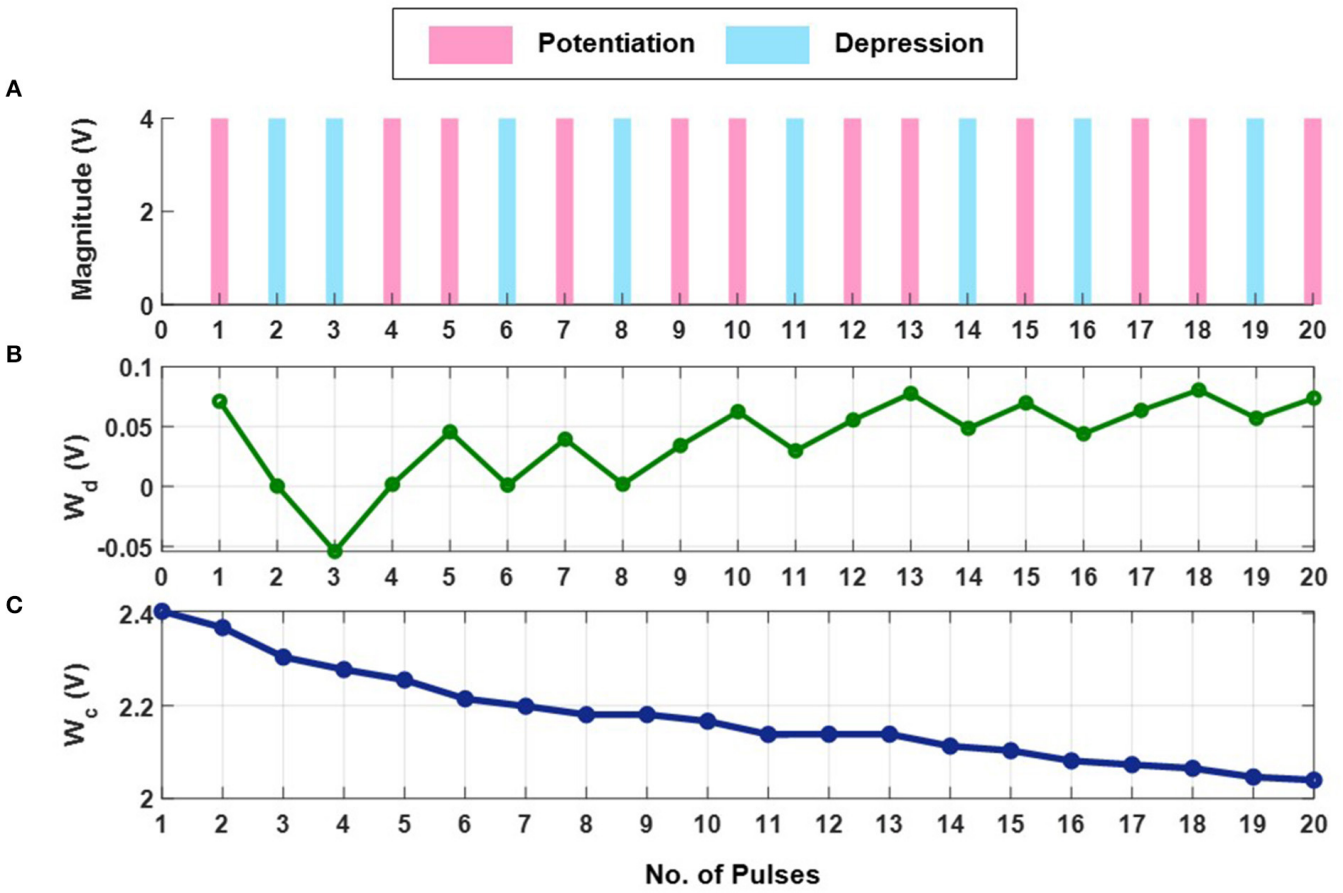
Experimental weight evolution of FN-synapse: **(A)** A random set of *potentiation* and *depression* pulses of equal magnitude and duration applied to the FN-synapse leading to **(B)** bidirectional evolution of weight (*W*_*d*_) and **(C)** the corresponding trajectory followed by the common-mode tunneling node (*W*_*c*_).

**Figure 3:**
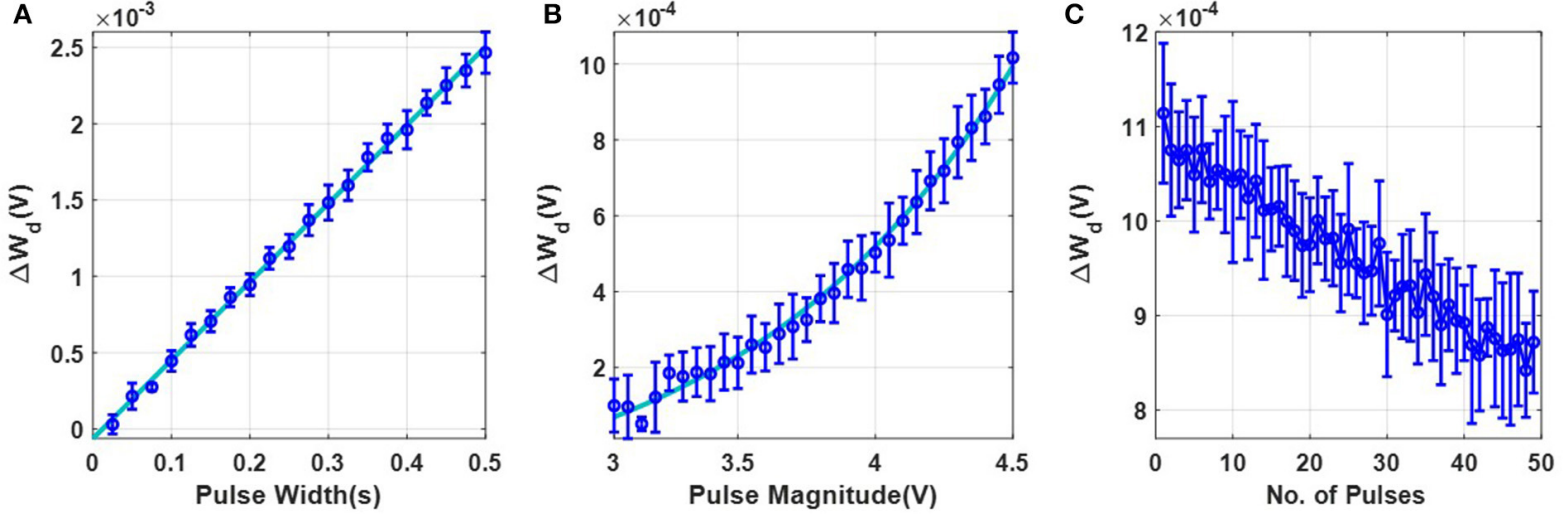
Experimental characterization of a single FN-synapse: **(A)** Dependence of change in magnitude of weight with change in pulse-width which follows a linear trajectory defined by *y* = *mx* + *c* (where *m* = 0.005136 and *c* = −6.227 × 10^−5^). **(B)** Dependence on pulse magnitude of the input pulse which follows an exponential trajectory defined by *y* = *c* × *exp*(*ax* + *b*) + *d* (where *a* = 1, *b* = −6.611, *c* = 0.009959 and *d* = −0.0002142). **(C)** Change in the magnitude of successive weight updates (Δ*W*_*d*_) corresponding to repeated stimulus.

The first set of experiments were designed to understand the *metaplasticity* exhibited by FN-synapses and how the synaptic weight and usage change in response to an external stimulation. The charge stored on the floating-gates in the FN-synapse were first initialized according to the procedure described in the Section 3 and in the Supplementary material. The tunneling barrier thickness in FN-synapse prototype shown in Figures 1D, E was chosen to be greater than 12 nm which makes the probability of direct-tunneling of electrons across the barrier to be negligible. The probability of FN-tunneling of electrons across the barrier (as shown in Figure 1F) is reduced to be negligible by lowering the electric potential of the tunneling nodes *W*^+^ and *W*^−^ (see Supplementary Figure 1) with respect to the reference ground to be less than 5 V. In this state the FN-synapse behaves as a standard non-volatile memory storing a weight proportional to $W_{d}=W^{+}-W^{-}$. To increase the magnitude of the stored weight a differential input pulse $\pm \frac{1}{2}X$ is applied across the capacitors that are coupled to the floating-gates (see Supplementary Figure 1). The electric potential of the floating-gate *W*^−^ is increased beyond 7.5 V where the FN-tunneling current *J*(*W*^−^) is significant. At the same time the electric potential of the floating-gate *W*^+^ is also pushed higher but *W*^−^ > *W*^+^ such that the FN-tunneling currents *J*(*W*^+^) < *J*(*W*^−^). As a result, the *W*^−^ node discharges at a rate that is faster than the *W*^+^ node. After the input pulse is removed, the potential of both *W*^−^ and *W*^+^ are pulled below 5 V and hence the FN-synapse returns to its non-volatile state. Figures 2A–C show the measured responses which shows that an FN-synapse can store both the weight and the usage history. When a series of *potentiation* and *depression* pulses of equal magnitude and duration is applied to the FN-synapse, as shown in Figure 2A, the weight stored *W*_*d*_ evolves bidirectionally (like a random walk) due to the input pulses (see Figure 2B). Meanwhile, the common-mode potential *W*_*c*_ decreases monotonically with the number of input pulses irrespective of the polarity of the input, as shown in Figure 2C. Therefore, *W*_*c*_ reliably tracks the usage history of the FN-synapse whereas *W*_*d*_ stores the weight of the synapse. Figures 3A, B show the measured weight update Δ*W*_*d*_ in response to different magnitudes and duration of the input pulses. For this experiment the common-mode $W_{c}=\frac{1}{2}\left(W^{+}+W^{-}\right)$ is held fixed. In Figure 3A, we can observe that for a fixed magnitude of input voltage pulses (= 4V) Δ*W*_*d*_ changes linearly with pulse width. Whereas, Figure 3B shows that the updated Δ*W*_*d*_ changes exponentially with respect to the magnitude of the input pulses (duration = 100 ms). Thus, the results show that pulse width modulation or pulse density modulation provides a more accurate control over the synaptic updates. Furthermore, in regard to energy dissipation per synaptic update pulse width modulation is also more attractive than using pulse magnitude variation. The energy required to write each time on FN-synapse can be estimated by measuring the energy drawn from the differential input source *X* in Supplementary Figure 1 to charge the coupling capacitor *C*_*c*_ and is given by


(4) $$\begin{array}{l} E_{write}=\frac{1}{2}C_{c}{\left(X\right)}^{2} \end{array}$$

This means that using smaller pulse magnitude accompanied by longer pulse width is preferable than the other way around in the context of write energy dissipation for the same desired change in weight. However, this would come at a cost of slower writing speed. Therefore, a trade-off exists. For the fabricated FN-synapse prototype, the magnitude of the coupling capacitor *C*_*c*_ is approximately 200f F which leads to 400f J for an input voltage pulse change of 2V across *C*_*c*_. For the differential input voltage pulse of 4V a total of 800f J of energy was dissipated for each potentiation and depression of the synaptic weights. When the common-mode *W*_*c*_ is not held fixed, irrespective of whether the weight *W*_*d*_ is increased or decreased (depending on the polarity of the input signal) the common-mode always decreases. Thus, *W*_*c*_ serve as an indicator of the usage of the synapse. Figure 3C shows the *metaplasticity* exhibited by an FN-synapse where we measured Δ*W*_*d*_ as a function of usage by applying successive *potentiation* input pulses of constant magnitude (4 V) and width (100 ms). Figure 3C shows that when the synapse is modulated with same excitation successively, the amount of weight update decreases monotonically with increasing usage, similar to the response illustrated in Figures 1C, F.

### 2.2. FN-synapse network capacity and memory lifetime without plasticity modulation

**Figure 4:**
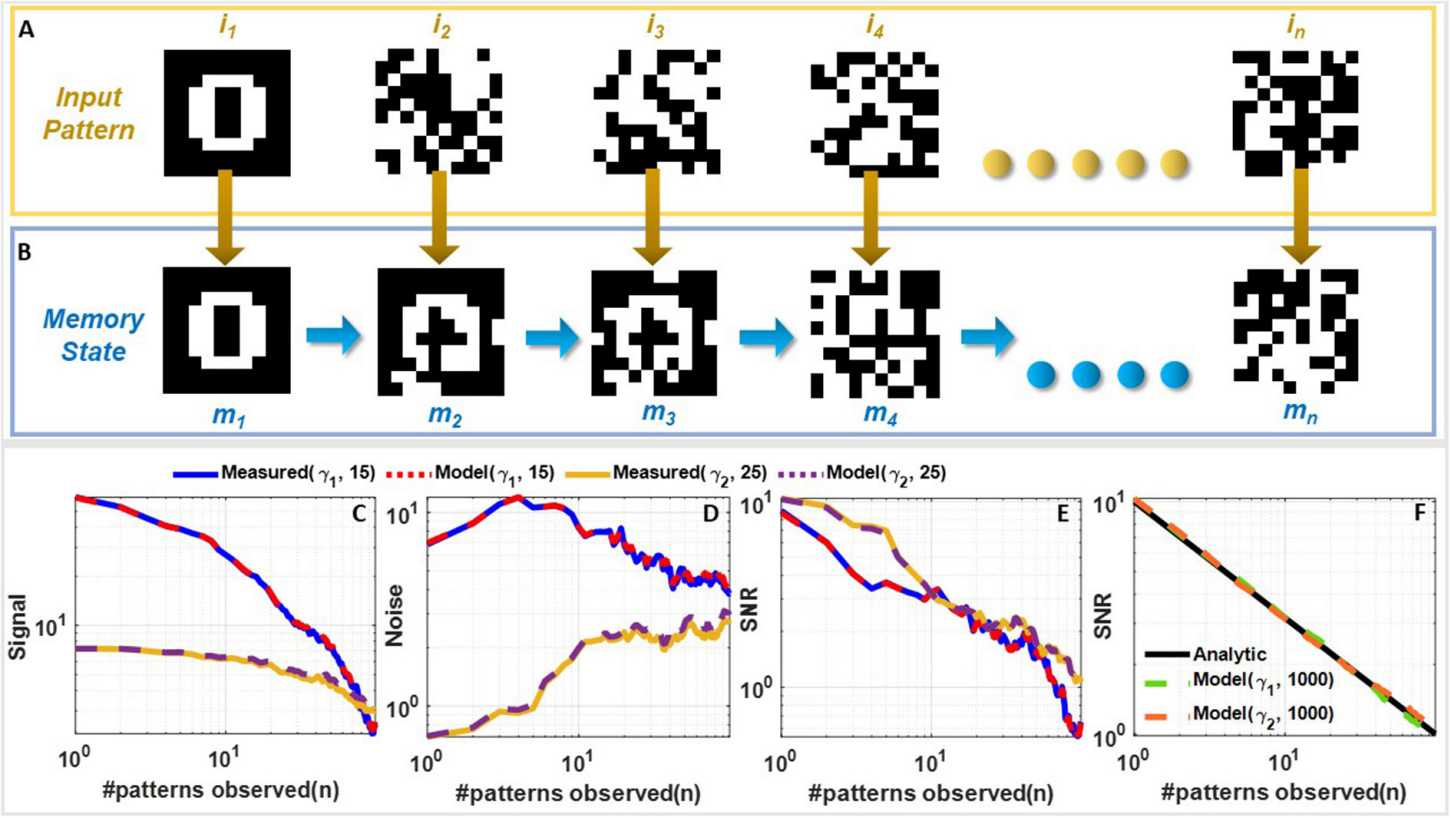
Comparison of measured and simulated memory consolidation for an empty FN-synapse network: **(A)** Set of 10 × 10 randomized noise inputs fed to a network of 100 FN-synapses initialized to store an image of the number 0 and **(B)** the corresponding memory evolution. Comparison of **(C)** signal strength, **(D)** noise strength, and **(E)** SNR for a network size of 100 synapse measured using the fabricated FN-synapse array shown in Figure 1F for 25 (for γ_1_) and 15 (for γ_2_) Monte-Carlo runs. **(F)** SNR comparison of the γ_1_ and γ_2_ models with the analytical model for 1,000 Monte Carlo simulations. The legends associated with the plots are specified as (γ, Number of Monte-Carlo runs). All of these results correspond to the behavior of an empty FN-synapse network.

The next set of experiments were designed to understand the FN-synaptic memory consolidation characteristics when the array is excited using a random binary input pattern (*potentiation* or *depression* pulses). This type of benchmark experiment is used extensively in memory consolidation studies (Benna and Fusi, 2016; Kirkpatrick et al., 2017) since analytical solutions exist for limiting cases which can be used to validate and compare the experimental results. A network comprising of *N* FN-synapses is first initialized to store zero weights (or equivalently *W*^−^ = *W*^+^). New memories were presented as random binary patterns (*N* dimensional random binary vector) that are applied to the *N* FN-synapses through either *potentiation* or *depression* pulses. Each synaptic element was provided with balanced input, i.e., equal number of *potentiation* and *depression* pulses. The goal of this experiment is to track the strength of a memory that is imprinted on this array in the presence of repeated new memory patterns. This is illustrated in Figures 4A, B where an initial input pattern (a 2D image of the number “0” comprising of 10 × 10 pixels) is written on a memory array. The array is then subjected to images of noise patterns that are statistically uncorrelated to the initial input pattern. It can be envisioned that as additional new patterns are written to the same array, the strength of a specific memory (of the image “0”) will degrade. Similar to the previous studies (Benna and Fusi, 2016; Kirkpatrick et al., 2017) we quantify this degradation in terms of signal-to-noise ratio (SNR). If *n* denotes the number of new memory patterns that have been applied to an empty FN-synapse array (initial weight stored on the network is zero), then the Section 3 shows that for the *p*^*th*^ update the retrieval memory signal *S*(*n, p*) power, the noise ν(*n, p*) power and the *SNR*(*n, p*) can be expressed analytically as


(5) $$\begin{array}{l} S^{2}\left(n,p\right)=\frac{1}{{\left(n+\gamma \right)}^{2}};\;\nu^{2}\left(n,p\right)=\frac{n}{N{\left(n+\gamma \right)}^{2}};\\ SNR\left(n,p\right)=\sqrt{\frac{N}{n}}. \end{array}$$

where γ>0 is a device parameter that depends on the initialization condition, material properties and duration of the input stimuli.

Equation (5) shows that the initial SNR is $\sqrt{N}$ and the SNR falls off according to a power-law decay with a slope of $\frac{1}{\sqrt{n}}$. Like previous consolidation studies (Benna and Fusi, 2016) we will assume that a specific memory pattern is retained as long as its SNR exceeds a predetermined threshold (unity in this experiment). Therefore, according to Equation (5) the network capacity and memory lifetime for FN-synapse scales linearly with the size of the network *N* when the initial weight across all synapse is zero. We verified the analytical expressions in Equation (5) for a network size of *N* = 100 using results measured from the FN-synapse chipset. Details of the hardware experiment is provided in the Section 3. Figures 4C–E show the retrieval signal, noise, and SNR obtained from the fabricated FN-synapse network for two different values of γ. We observe that the SNR obtained from the hardware results conform to the analytical expressions relatively well. The slight differences can be attributed to the Monte-Carlo simulation artifacts (only 25 and 15 iterations were carried out). In the Supplementary Figure 3, we show verification of these analytic expressions using a behavioral model of the FN-synapse which mimics the hardware prototype with great accuracy (as shown in Supplementary Figure 2). Details on the derivation of FN-synapse model is provided in the Section 3. The simulated results in Figures 4C–E verifies that results from the software model can accurately track the hardware FN-synapse measurements for both values of γ when subjected to the same stimuli. Therefore, FN-synapse and its behavioral model can be used interchangeably. The results in Figure 4F also show that when the number of iterations on the Monte-Carlo simulation is increased (1,000 iterations), the simulated SNR closely approximates the analytic expression. This verifies that hardware FN-synapse is also capable of exactly matching the optimal analytic consolidation characteristics. Figure 3C shows the measured evolution of weights stored in the FN-synapse where initially the weights grow quickly but after a certain number of updates settle to a steady value irrespective of new updates. This implies that the synapses have become rigid with an increase in its usage. This type of memory consolidation is also observed in EWC models which has been used for continual learning. However, note that unlike EWC models that need to store and update some measure of Fisher information, whereas, here the physics of the FN-synapse device itself can achieve similar memory consolidation without any additional computation.

### 2.3. Plasticity modulation of FN-synapse models

**Figure 5:**
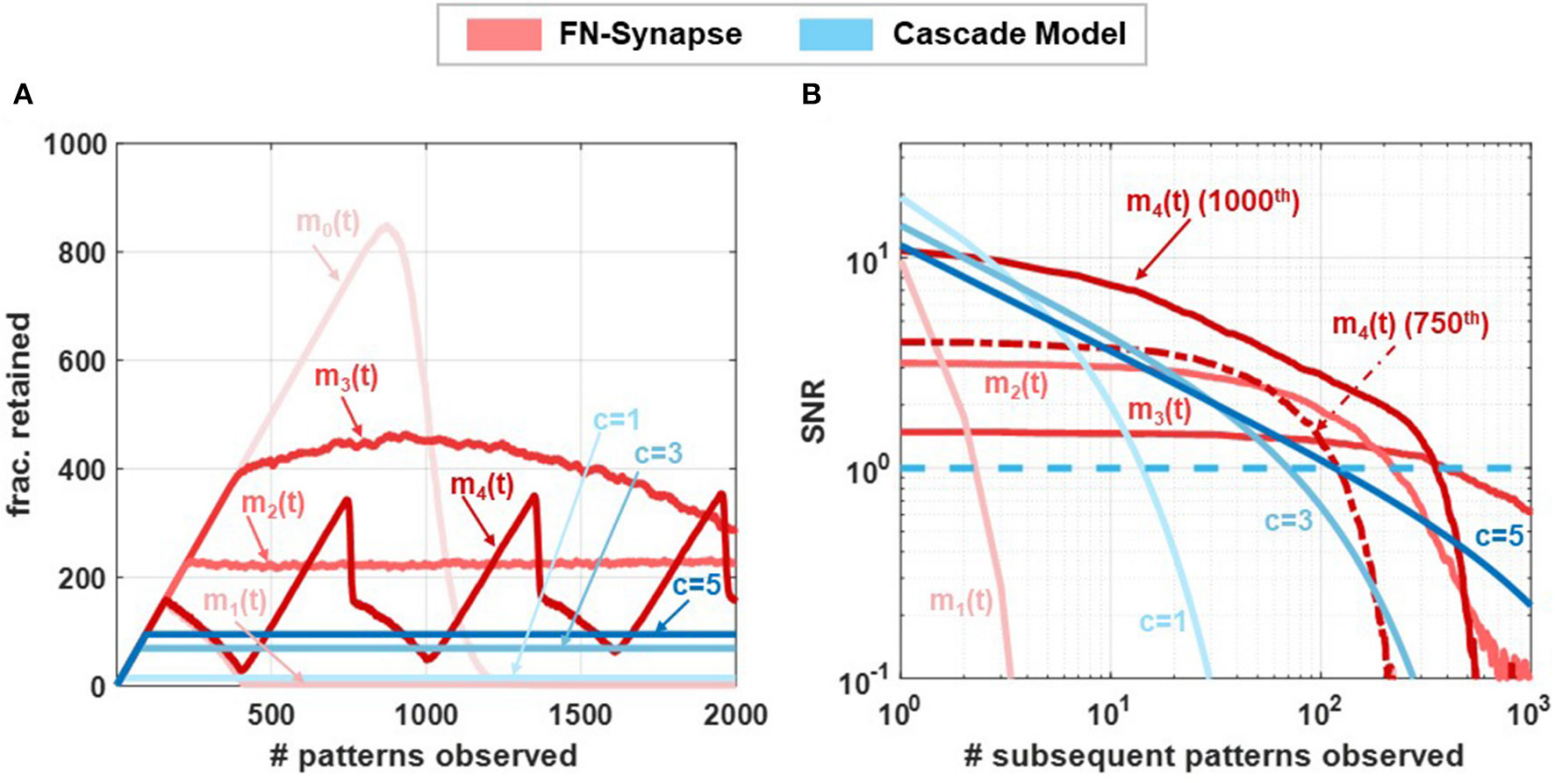
Network capacity and saturation experiments: Comparison of **(A)** no. of patterns retained by networks composed of 1,000 synapses following different synaptic models when exposed to 2,000 patterns and **(B)** steady-state SNR of the 1000^th^ update (*p* = 1, 000) of networks consisting of 1,000 synapses with various synaptic models when exposed to subsequent updates. For m_4(*t*)_ modulation SNR profiles for both 450^th^ and 1000^th^ (*p*= 450,1000) updates are shown.

In our next set of experiments, we verified that the plasticity of FN-synapses can be adjusted to mimic the consolidation properties of both EWC and steady-state models (such as cascade models). While the EWC model only allows for the retention of old memories, steady state/cascade models allow for both memory retention and forgetting. As a result, these models avoid *blackout catastrophe* whereas an EWC network is unable to retrieve any previous memories or store new experiences as the network approaches its capacity. Steady-state models allow the network to gracefully forget old memories and continue to remember new experiences *indefinitely*. For an FN-synapse network, a coupling capacitor in each synapse (shown in Supplementary Figure 1) which is driven by a global voltage signal *V*_*mod*_(*t*) (which produces $m\left(t\right)=\frac{dV_{mod}\left(t\right)}{dt}$) can control the plasticity of the FN-synapse to mimic the characteristics of a steady-state model. Details of the modified FN-synapse achieving a steady-state response are provided in the Section 3. To understand and compare the blackout catastrophe in FN-synapse models with a steady-state model, e.g., the cascade model we define the metric *#patterns*.*retained* as the total number of memory patterns whose SNR exceeds 1 at any given point of time. The *#patterns*.*retained* for FN-synapse network with modulation profiles *m*_0_(*t*), *m*_1_(*t*), *m*_2_(*t*), *m*_3_(*t*), and *m*_4_(*t*) of size *N* = 1, 000 is shown in Figure 5A together with those for cascade models of different levels of complexity (Benna and Fusi, 2016) (denoted by *c* = 1, .., 5). In order to calculate the *#patterns*.*retained* the SNR resulting from each stimulus was calculated and tracked at every observation to determine the number of such stimuli that had a corresponding SNR greater than unity. The profiles of *m*_1_(*t*), *m*_2_(*t*), and *m*_3_(*t*) are produced by changing *V*_*mod*_(*t*) at each update as three quarter, half, and quarter of the average of Δ*W*_*d*_ across all the synapses during the latest update, respectively, while *m*_0_(*t*) is achieved through a constant voltage signal *V*_*mod*_(*t*). We can observe in Figure 5A that the FN-synapse network with *m*_0_(*t*) forgets all observed patterns in addition to not forming any new memories as *#patterns*.*retained* goes to zero as the network capacity is reached starting from an empty network. Whereas, in the case for FN-synapse under *m*_1_(*t*) and *m*_2_(*t*) modulation profile the *#patterns*.*retained* reaches a finite value similar to that of the cascade models. This indicates that the FN-synapse network when subjected to plasticity modulation profiles continues to form new memory while gracefully forgetting the old ones. For the *m*_3_(*t*) modulation profile the network is slowly evolving and yet to reach the steady state condition within 2000^*th*^ update. The FN-synapse network under the *m*_4_(*t*) modulation profile, which switches between *m*_0_(*t*) and *m*_1_(*t*) periodically, is in an oscillatory steady-state with the same periodicity as the modulation profile itself. However, note that the network does not suffer from blackout catastrophe and has a variable capacity. This shows that the capacity of the FN-synapse network can also be tuned to the specificity of different applications. From the figure, we also observe that the steady state network capacity for *m*_2_(*t*) modulation profile is higher than that of cascade models. Note here that network capacity for cascade models may be increased by increasing the complexities of the synaptic model. Nevertheless, we find that network capacity for FN-synapse is comparable to cascade models of moderate complexities.

In order to understand the plasticity modulation further, we investigated the SNR for patterns introduced to a non-empty network. For this experiment, we tracked the 1000^*th*^ pattern observed by the network of *N* = 1, 000 synapse. Figure 5B shows the SNR of this pattern under *m*_1_(*t*)−*m*_4_(*t*) modulation profile along with cascade models of various complexity. Note that the x-axis now represents the age of the stimulus, i.e., number of patterns observed after the tracked pattern. For the modulation profile *m*_1_(*t*) the initial SNR is large, comparable to that of cascade models, but the SNR falls off quickly indicating high plasticity. Whereas, for modulation profile *m*_2_(*t*) and *m*_3_(*t*) the initial SNR is smaller than *m*_1_(*t*) but it falls off at a much later time similar to cascade models with high complexities. These SNR profiles for FN-synapse model with modulation *m*_1_(*t*)−*m*_3_(*t*) are similar to that of a constant weight decay synaptic model used in deep learning neural network as a regularization method. On the other hand, the SNR profile for the 1000^*th*^ pattern under *m*_4_(*t*) modulation has both high initial SNR and a large lifetime. However, from Figure 5B, we observe that the network is in an oscillatory state which indicates that this profile is specific to the 1000^*th*^ pattern, and if we tracked any other pattern the SNR profile would be different (for reference the SNR tracked for the 750^th^ update is also shown). This is not the case for the cascade models which would consistently have similar SNR profiles irrespective of the pattern that is tracked. Nevertheless, this SNR profile for the FN-synapse model would repeat itself corresponding to the periodicity of the modulation profile. This suggests that the amount of plasticity and memory lifetime for the FN-synapse model is readily tunable and depends on the amount of modulation provided to the network. We have also verified that the synaptic strength of FN-synapse is bounded similarly to that of the cascade models. This can be observed in Supplementary Figure 10 which shows that the variance in retrieval signal (Noise) of an FN-synapse network with both constant modulation and time-varying modulations remains bounded. Furthermore, Supplementary Figure 11 shows that plasticity modulation indeed introduces a forgetting mechanism as the SNR for different modulation profiles (when tracked from an empty network) starts to fall off earlier than the one without modulation. In addition to different modulation profile, the plasticity-lifetime tradeoff of the FN-synapse model can also be achieved by varying the parameter γ as shown in Supplementary Figure 12. Therefore, our synaptic models can exhibit memory consolidation properties similar to both EWC and steady-state models while being physically realizable and scalable for large networks.

### 2.4. Continual learning using FN-synapse

The next set of experiments was designed to evaluate the performance of FN-synapse neural network for a benchmark continual learning task. A fully-connected neural network with two hidden layers was trained sequentially on multiple supervised learning tasks. Details of the neural network architecture and training are given in Section 3 and in the Supplementary material. The network was trained on each task for a fixed number of epochs and after the completion of its training on a particular task *t*_*n*_, the dataset from *t*_*n*_ was not used for the successive task *t*_*n*+1_.

The aforementioned tasks were constructed from the Modified National Institute of Standards and Technology (MNIST) dataset, to address the problem of classifying handwritten digits in accordance with schemes popularly used in several continual-learning literature (Hsu et al., 2018). Also known as incremental domain learning using split-MNIST dataset, each task of this continual learning benchmark dictates the neural network to be trained as binary classifier which distinguishes between a set of two hand-written digits, i.e., the network is first trained to distinguish between the set [0, 1] as *t*_1_ and is then trained to distinguish between [2, 3] in *t*_2_, [4, 5] in *t*_3_, [6, 7] in *t*_4_, and [8, 9] in *t*_5_. Thus, the network acts as an even-odd number classifier during every task.

**Figure 6:**
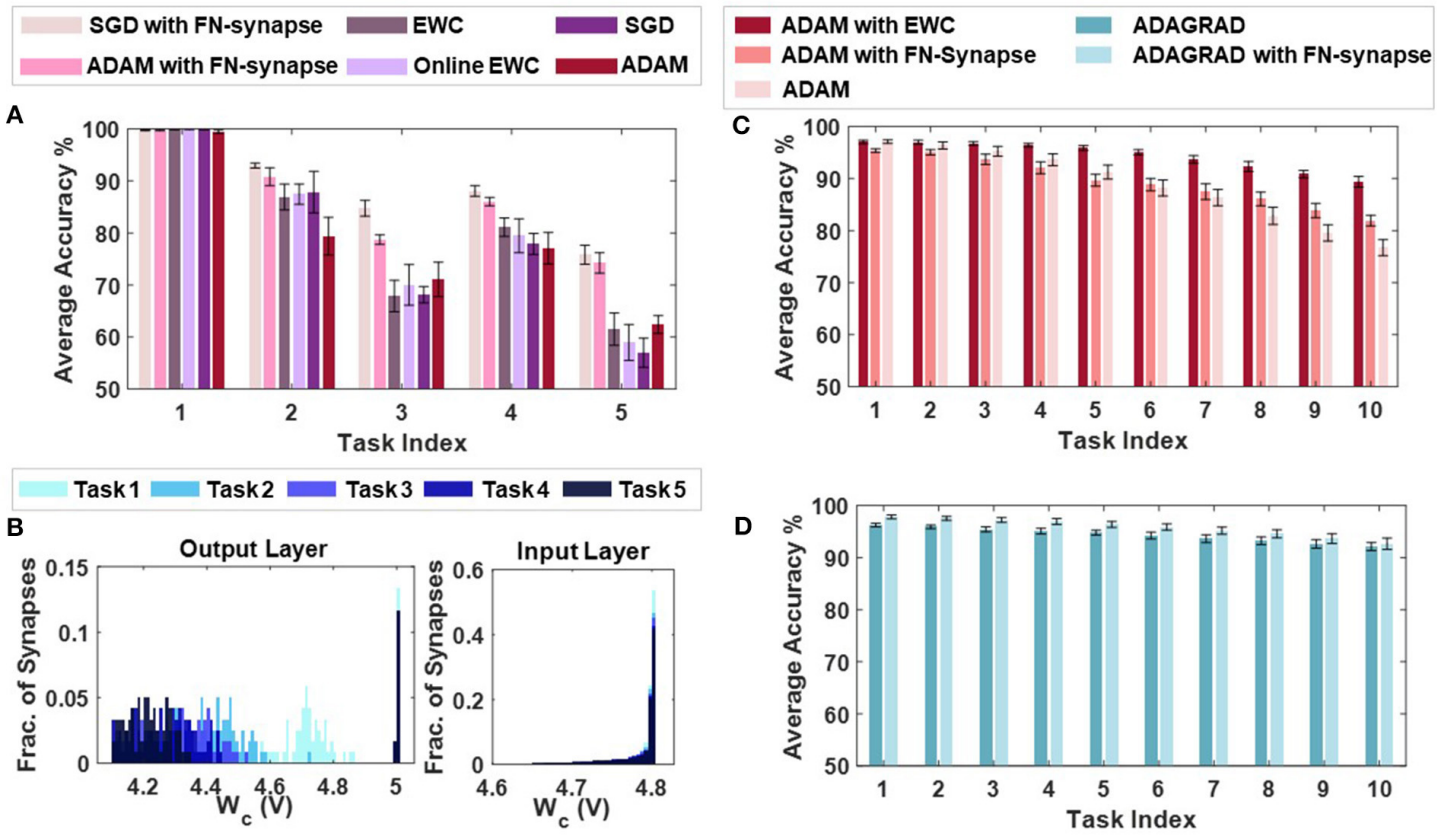
Continual learning benchmarks results and insights: **(A)** Overall average accuracy comparison of SGD and ADAM with FN-synapse, ADAM with EWC and Online EWC, SGD, and ADAM with conventional memory. **(B)** Distribution of the usage profile of weights in the output layer and the input layer of the FN-synapse neural network. Overall Average Accuracy comparison of incremental-domain learning scenarios on the Permuted MNIST dataset using **(C)** ADAM with EWC, ADAM with FN-Synapse and ADAM with conventional memory and **(D)** ADAGRAD with conventional memory and ADAGRAD with FN-synapse.

Supplementary Figures 7A–E compare the task-wise accuracy of networks trained with different learning and consolidation approaches. Note here that the absence of a data-point corresponding to a particular approach indicates that the accuracy obtained is below 50%. All the approaches taken into consideration perform equally well at learning *t*_1_ as illustrated in Supplementary Figure 7A. However, as the networks learn *t*_2_ (see Supplementary Figure 7B), the performance of both EWC (Kirkpatrick et al., 2017) and online EWC (Liu et al., 2018) degrade for task *t*_1_ as do the networks with conventional memory using SGD and ADAM. The FN-synapse based networks on the other hand retain the accuracy of task *t*_1_ far better in comparison. This advantage in retention comes at the cost of learning *t*_2_ marginally poorer than others. This trend of retaining the older memories or tasks far better than other approaches continues in successive tasks. Particularly, if we consider the retention of *t*_1_ when the networks are trained on *t*_3_ (see Supplementary Figure 7C), it can be observed that it is only the FN-synapse based networks that retain *t*_1_ while others fall below the 50% threshold. Similar trends can be observed in Supplementary Figures 7D, E. There are a few instances during the five tasks where the EWC variants and SGD with conventional memory marginally outperform or match the FN-synapse in terms of retention. However, if the overall average accuracy of all these approaches are compared (see Figure 6A), it is clearly evident that both the FN-synapse networks significantly outperform the others. It is also worth noting here that even when a network equipped with FN-synapse is trained using a computationally-inexpensive optimizer such as SGD, it shows remarkably superior performance than highly computationally-expensive approaches such as ADAM with conventional memory and ADAM with EWC variants.

The only drawback of the FN-synapse based approach is that its ability to learn the present task slightly degrades with every new task. This phenomenon results from the FN-synapses becoming more rigid and can be seen in Figure 6B which shows the evolution of plasticity of weights in the output and input layer of the network with successive tasks with respect to *W*_*c*_. As mentioned earlier, *W*_*c*_ keeps track of the importance of each weight as a function of the number of times it is used. The higher the *W*_*c*_ of a particular weight, the less it has been used and therefore, the more plastic it is and sensitive to change. On the other hand, a more rigid and frequently used weight has a lower value of *W*_*c*_. Suppose the output layer is considered from Figure 6B. In that case, it can be observed that with each successive task the *W*_*c*_ of the weights of the network collectively reduces, leading to more consolidation and consequently leaving the network with fewer plastic synapses to learn a new task. In comparison, the majority of the weights in the input layer remain relatively more plastic (or less spread out) owing to the redundancies in the network arising from the vanishing gradient problem (see Section 4 for more details). In Supplementary Figure 5, we show that the ability of the network to learn or forget new tasks is a function of the initial plasticity of the FN-synapses and can be readily adjusted.

In addition to the split-MNIST benchmark, the performance of FN-synapse based network was compared with EWC for the permuted MNIST benchmark. These incremental-domain learning experiments were carried out by randomly permuting the order of pixels of the images in the MNIST dataset in accordance with Hsu et al. (2018) to create new tasks. The overall average accuracy for 10 Monte Carlo simulations when using ADAM as the optimizer with EWC, FN-Synapse and conventional memory are depicted in Figure 6C. We can observe from Figure 6C that despite not being as retentive as EWC in this particular scenario, the network equipped with FN-synapse as the memory element performs better than the network without any memory consolidation mechanism, thereby exhibiting continual learning ability. Furthermore, when compared to a network with traditional memory employing an optimizer like ADAGRAD, which has been shown to be suitable for this learning scenario (Hsu et al., 2018), the FN-synapse network with ADAGRAD exhibits marginal improvements without any drop in performance with respect to the former as shown in Figure 6D.

## 3. Materials and methods

The main methods are described in this section of the paper while Supplementary material includes additional details, supporting information, and figures.

### 3.1. Weight update for differential synaptic model

Consider the differential synaptic model described by Figure 1C where the evolution of two dynamical systems with state variables *W*^+^ and *W*^−^ is governed by


(6) $$\begin{array}{l} \frac{dW^{+}}{dt}=-J\left(W^{+}\right)+\frac{1}{2}X\left(t\right)+\frac{1}{2}m\left(t\right) \end{array}$$

(7) $$\begin{array}{l} \frac{dW^{-}}{dt}=-J\left(W^{-}\right)-\frac{1}{2}X\left(t\right)+\frac{1}{2}m\left(t\right) \end{array}$$

where *J*(.) is an arbitrary function of the state variables, $+\frac{1}{2}X\left(t\right)$ or $-\frac{1}{2}X\left(t\right)$ are differential time varying inputs and *m*(*t*) is a common mode modulation input. In this differential architecture, we define the weight parameter *W*_*d*_ as $W_{d}=\frac{1}{2}\left(W^{+}-W^{-}\right)$ which represents the memory and the common-mode parameter *W*_*c*_ as $W_{c}=\frac{1}{2}\left(W^{+}+W^{-}\right)$ which represents the usage of the synapse. Applying this definition to (6) and (7), we obtain:


(8) $$\begin{array}{l} \frac{d\left(W_{c}+W_{d}\right)}{dt}=-J\left(W_{c}+W_{d}\right)+\frac{1}{2}X\left(t\right)+\frac{1}{2}m\left(t\right) \end{array}$$

(9) $$\begin{array}{l} \frac{d\left(W_{c}-W_{d}\right)}{dt}=-J\left(W_{c}-W_{d}\right)-\frac{1}{2}X\left(t\right)+\frac{1}{2}m\left(t\right) \end{array}$$

Now, adding and subtracting (8) and (9), we get:


(10) $$\begin{array}{l} \frac{dW_{c}}{dt}=-\left(\frac{J\left(W_{c}+W_{d}\right)+J\left(W_{c}-W_{d}\right)}{2}\right)+m\left(t\right) \end{array}$$

(11) $$\begin{array}{l} \frac{dW_{d}}{dt}=-\left(\frac{J\left(W_{c}+W_{d}\right)-J\left(W_{c}-W_{d}\right)}{2}\right)+X\left(t\right) \end{array}$$

Assuming that *W*_*c*_ >> *W*_*d*_, applying Taylor series expansion on (10) and (11) leads to


(12) $$\begin{array}{l} \frac{dW_{c}}{dt}=-J\left(W_{c}\right)+m\left(t\right) \end{array}$$

(13) $$\begin{array}{l} \frac{dW_{d}}{dt}=-J^{\prime }\left(W_{c}\right)W_{d}+X\left(t\right). \end{array}$$

This means that the modulation input impacts the usage of the synapse. Therefore, the plasticity of the synapse can be *tuned* using *m*(*t*) when needed. Now we first look into the trivial case when a constant modulation input is provided, i.e., *m*(*t*) = *c* where *c* is any arbitrary constant. In this scenario the plasticity of the synapse is solely dependent on the usage of the synapse as *m*(*t*) does not change with time. Substituting the derivative of *W*_*c*_ from (12), when *m*(*t*) is constant, into (13), the rate of change in *W*_*d*_ can be formulated as:


(14) $$\begin{array}{l} \frac{dW_{d}}{dt}=-\left[\frac{d^{2}W_{c}}{dt^{2}}{\left(\frac{dW_{c}}{dt}\right)}^{-1}\right]W_{d}+X\left(t\right) \end{array}$$

Please refer to the Supplementary material for detailed derivation. Equation (14) shows that the change in weight Δ*W*_*d*_ is directly proportional to the *curvature* of usage while being inversely proportional to the rate of usage.

### 3.2. Optimal usage profile

We define the decaying term in (14) as


(15) $$\begin{array}{l} r\left(t\right)=-\left[\frac{d^{2}W_{c}}{dt^{2}}{\left(\frac{dW_{c}}{dt}\right)}^{-1}\right] \end{array}$$

Now, comparing the weight update equation in (14) to the weight update equation for EWC in the balanced input scenario, the decay term has the following dependency with time for avoiding catastrophic forgetting.


(16) $$\begin{array}{l} r\left(t\right)=O\left(\frac{1}{t}\right) \end{array}$$

Now, the usage of a synapse is always monotonically increasing and since *W*_*c*_ represents the usage, it too needs to monotonic. At the same time *W*_*c*_ also needs to be bounded, therefore *W*_*c*_ has to monotonically decrease with increasing usage while satisfying the relationship in Equation (16). It can be shown that Equations (16) and (15) can be satisfied by any dynamical system of the form


(17) $$\begin{array}{l} W_{c}=\frac{1}{f\left(\log t\right)} \end{array}$$

where *f*(.) ≥ 0 is any monotonic function. Substituting Equation (17) in Equation (15) we obtain the corresponding usage profile as follows


(18) $$\begin{array}{l} r\left(t\right)=\frac{1}{t}\left(1+\frac{2f^{\prime }\left(\log t\right)}{\log t}-\frac{f^{\prime \prime }\left(\log t\right)}{f^{\prime }\left(\log t\right)}\right) \end{array}$$

where *f*′(log*t*) and *f*″(log*t*) are derivatives of *f*(log*t*) with respect to log*t*. While several choices of *f*(.) are possible, the simplest usage profile can be expressed as


(19) $$\begin{array}{l} W_{c}=\frac{\beta }{\log\left(t\right)} \end{array}$$

where β is any arbitrary constant. The corresponding non-linear function in this model is determined by substituting Equation (19) in Equation (12) to obtain


(20) $$\begin{array}{l} J\left(W_{c}\right)=\frac{1}{\beta }W_{c}^{2}\exp\left(-\frac{\beta }{W_{c}}\right). \end{array}$$

The expression for *J*(.) in Equation (20) bears similarity with the form of FN quantum-tunneling current (Lenzlinger and Snow, 1969) and Figures 1D–F show the realization of Equations (6) and (7) using FN tunneling junctions.

### 3.3. Achieving optimal usage profile on FN-synapse

For the differential FN tunneling junctions shown in Figure 1F and its equivalent circuit shown in the Supplementary Figure 1, the dynamical systems model is given by


(21) $$\begin{array}{l} C_{T}\frac{dW^{+}}{dt}=-J\left(W^{+}\right)+\frac{C_{c}}{2}\frac{dv_{in}}{dt} \end{array}$$

(22) $$\begin{array}{l} C_{T}\frac{dW^{-}}{dt}=-J\left(W^{-}\right)-\frac{C_{c}}{2}\frac{dv_{in}}{dt} \end{array}$$

where *W*^+^, *W*^−^ are the tunneling junction potentials, *C*_*c*_ is the input coupling capacitance, *v*_*in*_(*t*) is the input voltage to the coupling capacitance and *C*_*T*_ = *C*_*c*_ + *C*_*fg*_ is the total capacitance comprising of the coupling capacitance and the floating-gate capacitance *C*_*fg*_. *J*(.) are the FN tunneling currents given by


(23) $$\begin{array}{l} J\left(W^{+}\right)=\left(\frac{k_{1}}{k_{2}}\right){\left(W^{+}\right)}^{2}\exp\left(-\frac{k_{2}}{W^{+}}\right) \end{array}$$

(24) $$\begin{array}{l} J\left(W^{-}\right)=\left(\frac{k_{1}}{k_{2}}\right){\left(W^{-}\right)}^{2}\exp\left(-\frac{k_{2}}{W^{-}}\right) \end{array}$$

where *k*_1_ and *k*_2_ are device specific and fabrication specific parameters that remain relatively constant under isothermal conditions. Following the derivations in the previous sections and the expression in Equation (19) leads to a common-mode voltage *W*_*c*_ profile as


(25) $$\begin{array}{l} W_{c}\left(t\right)=\frac{k_{2}}{\log\left(k_{1}t+k_{0}\right)} \end{array}$$

where $k_{0}=\exp\left(\frac{k_{2}}{W_{c0}}\right)$ and *W*_*c*0_ refers to the initial voltage at the floating-gate.

### 3.4. FN-synpase network SNR estimation for random pattern experiment

Upon following the same procedure used in previous sections, the weight update equation for an FN-synapse using Equation (21) and Equation (22) can be expressed as


(26) $$\begin{array}{l} C_{T}\frac{dW_{d}}{dt}=-\left[\frac{d^{2}W_{c}}{dt^{2}}{\left(\frac{dW_{c}}{dt}\right)}^{-1}\right]W_{d}+C_{c}\frac{dv_{in}}{dt} \end{array}$$

We designed the floating-gate potential and the input voltage pulses such that the FN-dynamics is only active when there is an memory update. Therefore, the dynamics in Equation (26) evolve in a discrete manner with respect to the number of modulations. Assuming *C*_*T*_ = *C*_*c*_ we formulate a discretized version of the weight update dynamics from Equation (26) in accordance with the floating-gate potential profile of the device expressed in Equation (25) as follows


(27) $$\begin{array}{l} \begin{array}{c} \frac{△W_{d}\left(n\right)}{△t}=-k_{1}\left(1+\frac{2}{\log\left(k_{1}△tn+k_{0}\right)}\right)\left(\frac{1}{k_{1}△tn+k_{0}}\right)\\ W_{d}\left(n-1\right)+\frac{△v_{in}\left(n\right)}{△t} \end{array} \end{array}$$

(28) $$\begin{array}{l} \begin{array}{c} W_{d}\left(n\right)=\left[1-\left(1+\frac{2}{\log\left(k_{1}△tn+k_{0}\right)}\right)\left(\frac{1}{n+\frac{k_{0}}{k_{1}△t}}\right)\right]\\ W_{d}\left(n-1\right)+\left(v_{in}\left(n\right)-v_{in}\left(n-1\right)\right) \end{array} \end{array}$$

where *n* represents the number of patterns observed and Δ*t* is the duration of the input pulse. Let us denote the weight decay term as


(29) $$\begin{array}{l} \alpha \left(n\right)=\left[1-\left(1+\frac{2}{\log\left(k_{1}△tn+k_{0}\right)}\right)\left(\frac{1}{n+\frac{k_{0}}{k_{1}△t}}\right)\right] \end{array}$$

Thus, we obtain the weight update equation with respect to number of patterns observed as


(30) $$\begin{array}{l} W_{d}\left(n\right)=\alpha \left(n\right)W_{d}\left(n-1\right)+\left(v_{in}\left(n\right)-v_{in}\left(n-1\right)\right) \end{array}$$

When we start from an empty network, i.e., *W*_*d*_(0) = 0, the memory update can be expressed as a weighted sum over the past input as


(31) $$\begin{array}{l} \begin{array}{c} W_{d}\left(n\right)=\overset{n-2}{\underset{i=1}{\sum }}\left\{\left(\alpha \left(i+1\right)-1\right)\left(\overset{n}{\underset{j=i+2}{\prod }}\alpha \left(j\right)\right)v_{in}\left(i\right)\right\}\\ +\left(\alpha \left(n\right)-1\right)v_{in}\left(n-1\right)+v_{in}\left(n\right) \end{array} \end{array}$$

We define the retrieval signal and the noise associated with it as per the definition in Benna and Fusi (2016). For a network comprising of N synapses, each weight in the network is indexed as *W*_*d*_(*a, n*) where *a* = 1, ..., *N*. Similarly, the input applied to the *a*^*th*^ synapse after *n* patterns is *v*_*in*_(*a, n*). Then, the signal strength for the *p*^*th*^ update (where *p* < *n*) introduced to the initially empty network tracked after *n* patterns can be formulated as:


(32) $$\begin{array}{l} S\left(n,p\right)=\frac{1}{N}\langle \overset{N}{\underset{a=1}{\sum }}W_{d}\left(a,n\right)v_{in}\left(a,p\right)\rangle \end{array}$$

where angle brackets denote averaging over the ensemble of all of the input patterns seen by the network. If we assume that the input patterns are random binary events of ±1 and are uncorrelated between different synapses and memory patterns then substituting Equation (31) in Equation (32), we obtain


(33) $$\begin{array}{l} S\left(n,p\right)=\left(\alpha \left(p+1\right)-1\right)\overset{n}{\underset{j=p+2}{\prod }}\alpha \left(j\right) \end{array}$$

Given that in Equation (29), $k_{0}=O\left(10^{19}\right)$ and $k_{1}=O\left(10^{16}\right)$, the term $\left(1+\frac{2}{\ln\left(k_{1}△tn+k_{0}\right)}\right)\approx 1$, the signal power simplifies to:


(34) $$\begin{array}{l} S^{2}\left(n,p\right)=\frac{1}{{\left(n+\gamma \right)}^{2}} \end{array}$$

where $\gamma =\frac{k_{0}}{k_{1}△t}$ and depends on the pulse-width △*t* and the initial condition *k*_0_. The above equation shows that the signal's strength is a function of the system parameter γ and decays with the number of memory pattern observed. If we assume that the weight *W*_*d*_(*n*) is uncorrelated from the input *v*_*in*_(*n*) and that the inputs *v*_*in*_(1), *v*_*in*_(2), ...*v*_*in*_(*n*) are uncorrelated from each other, then the corresponding noise power is given by the variance of the retrieval signal expressed in Equation (32). This can be estimated as the sum of the power of all signals tracked at *n* except for the retrieval signal corresponding to the *p*^*th*^ update we are tracking and is given by:


(35) $$\begin{array}{l} \nu^{2}\left(n,p\right)=\frac{1}{N}\overset{n}{\underset{i=1,i\neq p}{\sum }}S^{2}\left(n,i\right) \end{array}$$

However, in order to derive a more tractable analytical expression for further analysis we added the retrieval signal as well into the summation which introduces a small error in the estimation (overestimating the noise by the retrieval signal term). This leads us to the following estimation of the noise power:


(36) $$\begin{array}{l} \nu^{2}\left(n,p\right)=\frac{n}{N{\left(n+\gamma \right)}^{2}} \end{array}$$

Based on the value of *n* in comparison to γ, we obtain two trends for the noise profile. When γ>>*n*,


(37) $$\begin{array}{l} \nu \left(n,p\right)=\frac{1}{\sqrt{N}}\left(\frac{\sqrt{n}}{\gamma }\right) \end{array}$$

which implies that noise increases with increase in updates initially. On the other hand, when γ << *n*,


(38) $$\begin{array}{l} \nu \left(n,p\right)=\frac{\sqrt{n}}{\sqrt{N}n}=\frac{1}{\sqrt{N}}\left(\frac{1}{\sqrt{n}}\right) \end{array}$$

which implies that noise falls with increase in updates in the later stages. The signal-to-noise ratio (SNR) of a network of size *N* can then be obtained as:


(39) $$\begin{array}{l} SNR\left(n,p\right)=\sqrt{\frac{S^{2}\left(n,p\right)}{\nu^{2}\left(n,p\right)}}=\sqrt{\frac{N}{n}} \end{array}$$

### 3.5. FN-synapse with tunable consolidation characteristics

In the previous sections, we derived the analytical expressions for the memory retrieval signal, the noise associated with it, and the corresponding SNR for the case when the modulation input *m*(*t*) was kept constant. This led to a synaptic memory consolidation which is similar to that of EWC. However, blackout catastrophic forgetting occurs in networks with such memory consolidation due to the absence of a balanced pattern retention and forgetting mechanism. The *forgetting* mechanism is naturally present in a steady state model such as the cascade model which do not suffer from memory “blackouts”. Since the increase in *retention* is equivalent to an increase in rigidity and *forgetting* is tantamount to a decrease in rigidity, it is necessary to adjust the plasticity/rigidity of the synapse accordingly. From Figures 2A, B, we notice that without external modulation *W*_*c*_ decreases monotonically with each new updates which correspondingly makes the synapse only rigid. Therefore, to balance the same, the idea is to keep *W*_*c*_ as steady as possible to keep the synapse plastic as long as possible by applying a modulation profile *m*(*t*) that *recovers/restores* *W*_*c*_ after every synaptic update. This results in *m*(*t*) of the form


(40) $$\begin{array}{l} m\left(t\right)=m\left(i\right)\delta \left(t-iT\right) \end{array}$$

where δ(*t*) is the Dirac-delta, *m*(*i*) is the magnitude of the modulation increment, and *T* is the time between each modulation increment. This increment is determined by the rate of the differential update to the FN-synapse. Integrating this form of *m*(*t*) into Equation (12) leads to


(41) $$\begin{array}{l} \frac{dW_{c}}{dt}=-J\left(W_{c}\right)+m\left(i\right)\delta \left(t-iT\right) \end{array}$$

which implies a tunable plasticity profile for the FN-synapse. An analytical solution to the differential equation (43) is difficult and hence we resort to a recursive solution. Due to the nature of the *m*(*t*), it can be seen that the initial condition of the variable *W*_*c*_ changes at increments of *T*, whereas between two modulation increments *W*_*c*_ evolves naturally according to Equation (25). Thus, the dynamics of *W*_*c*_ in the presence of the modulation increments can be described as


(42) $$\begin{array}{l} W_{c}(t)=\{\begin{array}{lll} W_{c0} & ; & t=0\\ W_{c}(t)+V_{mod}(t) & ; & t=iT\\ \frac{k_{2}}{\log(k_{1}(t-iT)+\exp(\frac{k_{2}}{W_{c}(iT)}))} & ; & iT<t<(i+1)T \end{array} \end{array}$$

where *V*_*mod*_(*t*) is an external voltage signal applied to the FN-synapse as shown in Supplementary Figure 1 and is given by:


(43) $$\begin{array}{l} V_{mod}\left(t\right)=\overset{\infty }{\underset{i=1}{\sum }}m\left(i\right)\delta \left(t-iT\right) \end{array}$$

In this case the change in plasticity of the synapse is determined by the step-size of the staircase voltage function *V*_*mod*_(*t*). Note that the weight update equation in (13) is still valid since *m*(*t*) is kept constant during differential input.

Although an analytic expression for the SNR is no longer tractable in this iterative form, the ability of the modulation term to regulate the plasticity and induce a more graceful form of forgetting is shown in the corresponding no. of patterns retained plot in Figure 5A and the SNR plot Figure 5B for various modulation input profiles.

### 3.6. Programming and initialization of FN-synapses

The potential corresponding to the tunneling nodes *W*^+^ and *W*^−^ can be accessed through a capacitively coupled node, as shown in Supplementary Figure 1. This configuration minimizes readout disturbances and the capacitive coupling also acts as a voltage divider so that the readout voltage is within the input dynamic range of the buffer. The configuration also prevents hot-electron injection of charge into the floating gate during readout operation. Details of initialization and programming are discussed in Mehta et al. (2020), so here we describe the methods specific for this work. The tunneling node potential was initialized at a specific region where FN-tunneling only occurs while there is a voltage pulse at the input node and the rest of the time it behaves as a non-volatile memory. This was achieved by first measuring the readout voltage every 1 s for a period of 5 min to ensure that the floating gate was not discharging naturally. During this period the noise floor of the readout voltage was measured to be ≈100μ*V*. At this stage, an voltage pulse of magnitude 1 V and duration 1 ms was applied at the input node and the change in readout voltage was measured. If the change was within the noise floor of the readout voltage, the potential of the tunneling nodes were increased by pumping electrons out of the floating gate using the program tunneling pin. This process involves gradually increasing the voltage at the program tunneling pin to 20.5 V (either from external source or from on-chip charge pump). The voltage at the program tunneling pin was held for a period of 30 s, after which it was set to 0 V. The process was repeated until substantial change in the readout voltage was observed (≈300μ*V*) after providing an input pulse. The readout voltage in this region was around 1.8 V.

### 3.7. Hardware and software experiments for random pattern updates

The fabricated prototype contained 128 differential FN tunneling junctions, which corresponds to 64 FN-synapses. However, due to the peripheral circuitry only one tunneling node could be accessed at a time for readout and modification. Now, since the memory pattern is completely random, each synapse can be modified independently without affecting the outcome of the experiment. Therefore, two tunneling nodes were initialized following the method described in the aforementioned section. Input pulses of magnitude 4 V and duration 100 ms was applied to both the tunneling nodes. The change in the readout voltages were measured, and the region where the update sizes of both the tunneling node would be equal was chosen as the initial zero memory point for the rest of the experiment. The nodes were then modified with a series of 100 *potentiation* and *depression* pulses of magnitude 4.5 V and duration 250 ms and the corresponding weights were recorded. This procedure represented the 100 updates of a single synapse. The tunneling nodes were then reinitialized to the zero memory point and the procedure was repeated with different random series of input pulses representing the modification of other 99 synapse in the network. The first input pulses of each series of modification forms the tracked memory pattern. To modify the value of γ the FN-synapses were initialized at a higher tunneling node potential.

The behavioral model of the FN-synapse was generated by extracting the device parameters *k*_1_ and *k*_2_ from the hardware prototype. The extracted parameters have been shown to capture the hardware response with an accuracy greater than 99.5% in our previous works (Zhou and Chakrabartty, 2017; Zhou et al., 2019). These extracted parameters were fed into a dynamical system which follows the usage profile described in the hardware implementation subsection and follow the weight update rule elaborated in the SNR estimation subsection to reliably imitate the behavior of the FN-synapse. The behavioral model network was started with exactly the same initial condition as hardware synapses and subjected to the exact memory patterns used for the hardware experiment for the same number of iterations. The simulation was also extended to 1,000 iterations and the corresponding responses are included in Figure 4F.

### 3.8. Probabilistic FN-synapse model

Adaption of FN-synapse occurs by tunneling of electrons through a triangular FN quantum-tunneling barrier. The tunneling current density is dependent on the barrier profile which in turn is a function of the floating-gate potential. When *W*^+^, *W*^−^ is around 7 V the synaptic update Δ*W*_*d*_ due to an external pulse can be determined by the continuous and deterministic form of the FN-synapse model (as described in the previous sections). Since the number of electrons tunneling across the barrier is relatively large (≫1), the method is adequate for determining Δ*W*_*d*_. However, once *W*^+^, *W*^−^ is around 6 V, each updates occurs due to the transport of a few electrons tunneling across the barrier and in the limit by a single electron tunneling across the barrier at a time. In this regime, the continuous behavioral model is no longer valid. Therefore, the behavioral model of the FN-synapse has to switch to a probabilistic model. In this mode of operation, we can assume that each electron tunneling event follows a Poisson process where the number of electrons *e*^+^(*n*), *e*^−^(*n*) tunneling across the two junctions during the *n*^*th*^ input pulse is estimated by sampling from a Poisson distribution with rate parameters λ^+^, λ^−^ given by


(44) $$\begin{array}{l} \lambda^{+}\left(n\right)=\frac{AJ\left(W^{+}\left(n\right)\right)}{q} \end{array}$$

(45) $$\begin{array}{l} \lambda^{-}\left(n\right)=\frac{AJ\left(W^{-}\left(n\right)\right)}{q}. \end{array}$$

*q* is the charge of an electron, *A* is the cross-sectional area of the tunneling junction. Using the sampled values of *e*^+^(*n*), *e*^−^(*n*), the corresponding discrete-time stochastic equation governing the dynamics of the tunneling node potentials *W*^+^(*n*), *W*^−^(*n*) is given by


(46) $$\begin{array}{l} W^{+}\left(n\right)=W^{+}\left(n-1\right)-\frac{qe^{+}\left(n\right)}{C_{T}} \end{array}$$

(47) $$\begin{array}{l} W^{-}\left(n\right)=W^{-}\left(n-1\right)-\frac{qe^{-}\left(n\right)}{C_{T}} \end{array}$$

where *C*_*T*_ is the equivalent capacitance of the tunneling node.

We have verified the validity/accuracy of the probabilistic model against the continuous-time deterministic model in high tunneling rate regimes. Supplementary Figure 4A shows that the output of the probabilistic model matches closely to the deterministic model and the deviation which arises due to the random nature of the probabilistic updates (shown in Supplementary Figure 4B) is within 200μ*V*. Using the probabilistic model we performed the memory retention and network capacity experiments (as discussed in the main manuscript) by initializing the tunneling nodes at a low potential. In this regime, each updates to the FN synapse results from tunneling of a few electrons. Supplementary Figures 4C, D show that even when each update sizes are on the order of tens of electrons, the network capacity and memory retention time remains unaffected. However, as the synaptic voltage is modified by less than ten electrons per update (shown in Supplementary Figure 4E), the SNR curve starts to shift downwards and the network capacity along with memory retention time decreases. The tunneling node potential can be pushed further down to a region where the synapses might not even register modifications at times and other times update sizes drop down to single electron per modification (see Supplementary Figure 4F). In this regime, the SNR curve shifts down further, the SNR decay still obeys the power-law curve.

### 3.9. Neural network implementation using FN-synapses

The MNIST dataset was split into 60,000 training images and 10,000 test images which yielded about 6,000 training images and 1,000 test images per digit. Each image, originally of 28 × 28 pixels, was converted to 32 × 32 pixels through zero-padding. This was followed by standard normalization to zero mean with unit variance. The code for implementing the non-FN-synapse approaches such as EWC and online EWC were obtained from the repository mentioned in Hsu et al. (2018). To enforce an equitable comparison, the same neural network architecture (as shown in Supplementary Figure 6), in the form a multi-layered perceptron (MLP) with an input layer of 1024 nodes, two hidden layers of 400 nodes each (paired with the ReLU activation function) and a softmax output layer of 2 nodes, has been utilized by every method mentioned in this work. Based on the optimizer in use, a learning rate of 0.001 was chosen for both SGD and ADAM (with additional parameters β_1_, β_2_, and ϵ set to 0.9, 0.999, and 10^−8^, respectively, for the latter). Each model was trained with a mini-batch size of 128 for a period of 4 epochs.

Similar to the continual learning experiments conducted on split-MNIST, benchmark incremental-domain learning experiments were also carried out by randomly permuting the order of pixels of the images in the MNIST dataset in accordance with Hsu et al. (2018) which is referred as the Permuted-MNIST. The architecture of the neural network employed is similar to the one for the split-MNIST with the exception of being equipped with 1,000 neurons in each of the two hidden layers instead of 400 and with 10 neurons in the output layer instead of 2. This essentially means that at each task, the network learns a new set of permutations of the 10 digits. The network was trained on 10 such tasks for 3 epochs using a learning rate of 0.0001 for ADAM and 0.001 for ADAGRAD.

Corresponding to every weight/bias in the MLP, an instance of the FN-synapse model was created and initialized to a tunneling region according to the initial *W*_*c*_ value. As demonstrated by the measured results, Δ*W*_*d*_ can be modulated linearly and precisely by changing the pulse-width of the *potentiation*/*depression* pulses. Therefore, each weight update (calculated according to the optimizer in use) is mapped as an input pulse of proportional duration for the FN synapse instance. Then, every instance of the FN-synapse model is updated according to Equation (27) and the *W*_*d*_ thus obtained in voltage is scaled back to a unit-less value and within the required range of the network.

## 4. Discussion

In this paper, we reported a differential FN quantum-tunneling based synaptic device that can exhibit near-optimal memory consolidation that has been previously demonstrated using only algorithmic models. The device called FN-synpase, like its algorithmic counterparts, stores the value of the weight and a relative usage of the weight that determines the plasticity of the synapse. Similar to algorithmic consolidation models, an FN-synapse, “protects” important memory by reducing the plasticity of the synapse according to its usage for a specific task. However, unlike its algorithmic counterparts like the cascade or EWC models, the FN-Synapse doesn't require any additional computational or storage resources. In EWC models memory consolidation in continual learning is achieved by augmenting the loss function using penalty terms that are associated with either Fisher information (Kirkpatrick et al., 2017) or the historical trajectory of the parameter over the course of learning (Chaudhry et al., 2018; Liu et al., 2018). Thus, the synaptic updates require additional pre-processing of the gradients, which in some cases could be computationally and resource intensive. FN-synapse on the other hand, does not require any pre-processing of gradients and instead can exploit the physics of the device itself for synaptic intelligence and for continual learning. For some benchmark tasks, we have shown an FN-synapse network shows better multi-task accuracy compared to other continual learning approaches. This leads to the possibility that the intrinsic dynamics of the FN-synapse could provide important clues on how to improve the accuracy of other continual learning models as well.

Figures 6A, B also show the importance of the learning algorithm in fully exploiting the available network capacity. While the entropy of the FN-synapse weights for the output layer is relatively high, the entropy of the weights of the input layer is still relatively low, implying most of the input layer weights remain unused. This is an artifact of *vanishing gradients* in a standard backpropagation based neural network learning. Thus, it is possible that improved backpropagation algorithms (Deng et al., 2016; Tan and Lim, 2019) might be able to mitigate this artifact and in the process enhance the capacity and the performance of the FN-synapse network. In Supplementary Figure 8, we show that FN-synapse based neural network is able to maintain its performance even when the network size is increased. Thus, it is possible that the network becomes capable of learning more complex tasks due to increase in overall plasticity of the network while ensuring considerably better retention than neural networks with traditional synapses.

In addition to being physically realizable, the FN-synapse implementation also allows interpolation between a steady state consolidation model and the EWC consolidation models. This is important because it is widely accepted that the EWC model can potentially suffer from blackout catastrophe (Kirkpatrick et al., 2017) as the learning network approaches its capacity. During this phase, the network becomes incapable of retrieving any previous memory as well as is unable to learn new ones (Kirkpatrick et al., 2017). Steady-state models such as the cascade consolidation models and SGD-based continuous learning models avoid this catastrophe by gracefully forgetting old memories. As shown in Figure 5A, an FN-synapse network, through the use of a global modulation factor *m*(*t*), is able to interpolate between the two models. In fact, the results in Figures 5A, B, show that the number of patterns/memories retained in an FN-synapse network under modulation profile *m*_2_(*t*) at steady state is higher compared to that of a high-complexity cascade model for a network size of *N* = 1, 000 synapses. Even though we have not used the interpolation feature for benchmark experiments, we believe that this attribute is going to provide significant improvements for continuous learning of a large number of tasks.

The interpolation property of FN-synapse could mimic some attributes of *metaplasticity* observed in biological synapses and dendritic spines (Mahajan and Nadkarni, 2019). The role of metaplasticity, the second-order plasticity of a synapse which assigns a task-specific importance to every successive task being learned (Laborieux et al., 2021), is widely accepted as the fundamental component of neural processes key to memory and learning in the hippocampus (Abraham and Bear, 1996; Abraham, 2008). Since unregulated plasticity leads to runaway effects resulting in previously stored memories to be impaired at saturation of synaptic strength (Brun et al., 2001), metaplasticity serves as a regulatory mechanism which dynamically links the history of neuronal activity with the current response (Hulme et al., 2014). The FN-synapse mimics the same regulatory mechanism through the decaying term *r*(*t*) that takes into account the history of usage or neuronal activity to determine the plasticity of the synapse for future use as well as prevents runaway effects by making the synapses rigid at saturation.

The on-device memory consolidation in FN-synapse can not only minimize the energy requirements in continual learning tasks, additionally, the energy required for a single synaptic weight update is also lower than memristor-based synaptic updates for a fixed precision of update. This attribute has been validated in our previous works (Mehta et al., 2022) where the update energy was estimated to be as low as 5f J increasing up to 2.5p J depending on the status of the FN-synapse and the desired change in synaptic weights. Note that the energy required to change the synaptic weight is derived from the FN-tunneling current and not from the electrostatic energy used for charging the coupling capacitor. Thus, by designing more efficient charge-sharing techniques across the coupling capacitors the energy-efficiency of FN-synaptic updates can be significantly improved. Furthermore, when implemented on more advanced silicon process nodes, the capacitances could be scaled which can improve the energy-efficiency of FN-synapse by an order of magnitude. Compared to memristor-based synapses, the FN-synapse can also exhibit high endurance 10^6^−10^7^ cycles without any deterioration. However, the key distinction lies in terms of the dynamic range of the stored weights. Generally, a single memristor has two distinct conductive states (corresponding to “0” or “1”) which give each device a 1-bit resolution. When used in a crossbar array, highly-dense designs can reach densities up to 76.5 *nm*^2^ per bit as reported by Poddar et al. (2021) where a 3-D memristor array was constructed using Perovskite quantum wires. The dynamic range or resolution of such designs is determined by the number of memristive devices that can be packed into the smallest feasible physical form factor. If we consider multi-level memristors instead, the resolution per memristor can reach up to 3-5 bits depending on the number of stable distinguishable conductive states (He et al., 2017; Wu et al., 2019; Lee et al., 2021). In comparison, the dynamic range of the FN-synapse (a single device) is considerably higher as it is determined by the number of electrons stored on the floating-gates which in-turn is determined by the FN-synapse form-factor and the dielectric property of the tunneling barrier. Thus, theoretically, the dynamic range and the operational-life of the FN-synapse seems to be constrained by the single-electron quantization. However, at low-tunneling regimes, the transport of single electrons across the tunneling barrier becomes probabilistic where the probability of tunneling is now modulated by the external signals *X*(*t*) and *m*(*t*). In the Section 3 and in Supplementary Figure 4, we show that a stochastic dynamical system model emulating the single-electron dynamics in the FN-synapse can produce $O\left(1/\sqrt{t}\right)$ consolidation characteristics for the benchmark random input patterns experiment for an empty network. The SNR still follows the power-law curve and the FN-synapse network continues to learn new experiences even if the synaptic updates are based on discrete single-electron transport. A more pragmatic challenge in using the FN-synapse will be the ability of the read-out circuitry to discriminate between the changes in floating-gate voltage due to single-electron tunneling events. For the magnitude of the floating-gate capacitance, the change in voltage would be in the order of 100 nV per tunneling event. A more realistic scenario would be to measure the change in voltage after 1,000 electron tunneling events which would imply measuring 100 μV changes. Although this will reduce the resolution of the stored weights/updates to 14 bits, recent studies have shown that neural networks with training precisions as low as 8 bits (Sun et al., 2019) and networks with inference precisions as low as 2–4 bits (Choi et al., 2018, 2019) are often capable of exhibiting remarkably good learning abilities. In Supplementary Figure 9, we show that for the split-MNIST task, the performance of the FN-synapse based neural network remains robust even in the presence of 5% device mismatch.

Another point of discussion is whether the optimal decay profile r(t)≈O(1/t) can be implemented by other synaptic devices, in particular, the energy-efficient memristor-based synapses that have been proposed for neuromorphic computing (Tuma et al., 2016; Fuller et al., 2019; Pal et al., 2019a,b; Karunaratne et al., 2020; Mehonic et al., 2020). Recent works using memristive devices have demonstrated on-device *metaplasticity* (Giotis et al., 2022), however, achieving an optimal decay profile would require additional control circuitry, storage and read-out circuits. In this regard, we believe that the FN-synapse represents one of the few, if not the only class of synaptic devices that can achieve optimal memory consolidation on a single device.

## Data availability statement

The raw data supporting the conclusions of this article will be made available by the authors, without undue reservation.

## Author contributions

SC and MR came up with the concept of FN-synapse. MR, SB, and SC designed the hardware and simulation experiments. MR designed the 64 element FN-synapse chipset. MR and SB conducted the simulation and hardware experiments. SC provided supervision on all tasks. All authors contributed toward writing and proof-reading the manuscript. All authors contributed to the article and approved the submitted version.

## References

[B1] Abraham W. C. (2008). Metaplasticity: tuning synapses and networks for plasticity. Nat. Rev. Neurosci. 9, 387–387. 10.1038/nrn2356 18401345

[B2] Abraham W. C. Bear M. F. (1996). Metaplasticity: the plasticity of synaptic plasticity. Trends Neurosci. 19, 126–130. 10.1016/S0166-2236(96)80018-X 8658594

[B3] Aljundi R. Babiloni F. Elhoseiny M. Rohrbach M. Tuytelaars T. (2017). Memory aware synapses: learning what (not) to forget. arXiv preprint arXiv: 1711.09601. 10.48550/arXiv.1711.09601

[B4] Amit D. J. Fusi S. (1994). Learning in neural networks with material synapses. Neural Comput. 6, 957–982. 10.1162/neco.1994.6.5.957

[B5] Benna M. Fusi S. (2016). Computational principles of synaptic memory consolidation. Nat. Neurosci. 19, 1697–1706. 10.1038/nn.4401 27694992

[B6] Brun V. H. Ytterbø K. Morris R. G. Moser M.-B. Moser E. I. (2001). Retrograde amnesia for spatial memory induced by NMDA receptor-mediated long-term potentiation. J. Neurosci. 21, 356–362. 10.1523/JNEUROSCI.21-01-00356.2001 11150353 PMC6762446

[B7] Chaudhry A. Dokania P. K. Ajanthan T. Torr P. H. S. (2018). Riemannian walk for incremental learning: understanding forgetting and intransigence. arXiv preprint arXiv: 1801.10112. 10.1007/978-3-030-01252-6_33

[B8] Choi J. Venkataramani S. Srinivasan V. V. Gopalakrishnan K. Wang Z. Chuang P. (2019). Accurate and efficient 2-bit quantized neural networks. Proc. Mach. Learn. Syst. 1, 348–359.

[B9] Choi J. Wang Z. Venkataramani S. Chuang P. I. Srinivasan V. Gopalakrishnan K. (2018). PACT: parameterized clipping activation for quantized neural networks. arXiv preprint arXiv: 1805.06085. 10.48550/arXiv.1805.06085

[B10] Deng Y. Bao F. Kong Y. Ren Z. Dai Q. (2016). Deep direct reinforcement learning for financial signal representation and trading. IEEE Trans. Neural Netw. Learn. Syst. 28, 653–664. 10.1109/TNNLS.2016.2522401 26890927

[B11] Fuller E. J. Keene S. T. Melianas A. Wang Z. Agarwal S. Li Y.. (2019). Parallel programming of an ionic floating-gate memory array for scalable neuromorphic computing. Science 364, 570–574. 10.1126/science.aaw5581 31023890

[B12] Fusi S. (2002). Hebbian spike-driven synaptic plasticity for learning patterns of mean firing rates. Biol. Cybernet. 87, 459–470. 10.1007/s00422-002-0356-8 12461635

[B13] Fusi S. Abbott L. (2007). Limits on the memory storage capacity of bounded synapses. Nat. Neurosci. 10, 485–493. 10.1038/nn1859 17351638

[B14] Fusi S. Drew P. J. Abbott L. (2005). Cascade models of synaptically stored memories. Neuron 45, 599–611. 10.1016/j.neuron.2005.02.001 15721245

[B15] Giotis C. Serb A. Manouras V. Stathopoulos S. Prodromakis T. (2022). Palimpsest memories stored in memristive synapses. Sci. Adv. 8, eabn7920. 10.1126/sciadv.abn7920 35731877 PMC9217086

[B16] He W. Sun H. Zhou Y. Lu K. Xue K. Miao X. (2017). Customized binary and multi-level HfO2- x-based memristors tuned by oxidation conditions. Sci. Rep. 7, 1–9. 10.1038/s41598-017-09413-9 28855562 PMC5577168

[B17] Hsu Y.-C. Liu Y.-C. Ramasamy A. Kira Z. (2018). Re-evaluating continual learning scenarios: a categorization and case for strong baselines. arXiv preprint arXiv: 1810.12488. 10.48550/arXiv.1810.12488

[B18] Hulme S. R. Jones O. D. Raymond C. R. Sah P. Abraham W. C. (2014). Mechanisms of heterosynaptic metaplasticity. Philos. Trans. R. Soc. B Biol. Sci. 369, 20130148. 10.1098/rstb.2013.0148 24298150 PMC3843880

[B19] Karunaratne G. Le Gallo M. Cherubini G. Benini L. Rahimi A. Sebastian A. (2020). In-memory hyperdimensional computing. Nat. Electron. 3, 327–337. 10.1038/s41928-020-0410-3

[B20] Kirkpatrick J. Pascanu R. Rabinowitz N. Veness J. Desjardins G. Rusu A. A.. (2017). Overcoming catastrophic forgetting in neural networks. Proc. Natl. Acad. Sci. U.S.A. 114, 3521–3526. 10.1073/pnas.1611835114 28292907 PMC5380101

[B21] Laborieux A. Ernoult M. Hirtzlin T. Querlioz D. (2021). Synaptic metaplasticity in binarized neural networks. Nat. Commun. 12, 1–12. 10.1038/s41467-021-22768-y 33953183 PMC8100137

[B22] Lee S. Jeon J. Eom K. Jeong C. Yang Y. Park J.-Y.. (2021). Multi-level memristors based on two-dimensional electron gases in oxide heterostructures for high precision neuromorphic computing. Res. Square. 10.21203/rs.3.rs-1019162/v1

[B23] Lee S. Kim J. Ha J. Zhang B. (2017). Overcoming catastrophic forgetting by incremental moment matching. arXiv preprint arXiv: 1703.08475. 10.48550/arXiv.1703.08475

[B24] Lenzlinger M. Snow E. H. (1969). Fowler–Nordheim tunneling into thermally grown SiO2. J. Appl. Phys. 40, 278–283. 10.1063/1.1657043

[B25] Li Q. Navakkode S. Rothkegel M. Soong T. W. Sajikumar S. Korte M. (2017). Metaplasticity mechanisms restore plasticity and associativity in an animal model of Alzheimer's disease. Proc. Natl. Acad. Sci. U.S.A. 114, 5527–5532. 10.1073/pnas.1613700114 28484012 PMC5448214

[B26] Liu X. Masana M. Herranz L. van de Weijer J. López A. M. Bagdanov A. D. (2018). Rotate your networks: better weight consolidation and less catastrophic forgetting. arXiv preprint arXiv: 1802.02950. 10.1109/ICPR.2018.8545895

[B27] Mahajan G. Nadkarni S. (2019). Intracellular calcium stores mediate metaplasticity at hippocampal dendritic spines. J. Physiol. 597, 3473–3502. 10.1113/JP277726 31099020 PMC6636706

[B28] Mehonic A. Sebastian A. Rajendran B. Simeone O. Vasilaki E. Kenyon A. J. (2020). Memristors–from in-memory computing, deep learning acceleration, and spiking neural networks to the future of neuromorphic and bio-inspired computing. Adv. Intell. Syst. 2, 2000085. 10.1002/aisy.202000085

[B29] Mehta D. Aono K. Chakrabartty S. (2020). A self-powered analog sensor-data-logging device based on Fowler-Nordheim dynamical systems. Nat. Commun. 11. 10.1038/s41467-020-19292-w 33116118 PMC7595237

[B30] Mehta D. Rahman M. Aono K. Chakrabartty S. (2022). An adaptive synaptic array using Fowler–Nordheim dynamic analog memory. Nat. Commun. 13, 1–11. 10.1038/s41467-022-29320-6 35351886 PMC8964701

[B31] Pal S. Bose S. Islam A. (2019a). Design of memristor based low power and highly reliable reram cell. Microsyst. Technol. 28, 1–15. 10.1007/s00542-019-04582-1

[B32] Pal S. Bose S. Ki W.-H. Islam A. (2019b). Design of power-and variability-aware nonvolatile rRAM cell using memristor as a memory element. IEEE J. Electron Devices Soc. 7, 701–709. 10.1109/JEDS.2019.2928830

[B33] Poddar S. Zhang Y. Gu L. Zhang D. Zhang Q. Yan S.. (2021). Down-scalable and ultra-fast memristors with ultra-high density three-dimensional arrays of perovskite quantum wires. Nano Lett. 21, 5036–5044. 10.1021/acs.nanolett.1c00834 34124910

[B34] Rahman M. Zhou L. Chakrabartty S. (2022). SpotKD: a protocol for symmetric key distribution over public channels using self-powered timekeeping devices. IEEE Trans. Inform. Forensics Sec. 17, 1159–1171. 10.1109/TIFS.2022.3158089

[B35] Roxin A. Fusi S. (2013). Efficient partitioning of memory systems and its importance for memory consolidation. PLoS Comput. Biol. 9, e1003146. 10.1371/journal.pcbi.1003146 23935470 PMC3723499

[B36] Sohoni N. S. Aberger C. R. Leszczynski M. Zhang J. Re C. (2019). Low-memory neural network training: a technical report. arXiv preprint arXiv:1904.10631. 10.48550/arXiv.1904.10631

[B37] Sun X. Choi J. Chen C.-Y. Wang N. Venkataramani S. Srinivasan V. V.. (2019). “Hybrid 8-bit floating point (HFP8) training and inference for deep neural networks, in Advances in Neural Information Processing Systems, Vol. 32, eds Wallach H. Larochelle H. Beygelzimer A. dAlché-Buc F. Fox E. Garnett R. (Vancouver, CA: Curran Associates, Inc.).

[B38] Tan H. H. Lim K. H. (2019). “Vanishing gradient mitigation with deep learning neural network optimization, in 2019 7th International Conference on Smart Computing & Communications (ICSCC) (IEEE), 1–4. 10.1109/ICSCC.2019.8843652

[B39] Tuma T. Pantazi A. Le Gallo M. Sebastian A. Eleftheriou E. (2016). Stochastic phase-change neurons. Nat. Nanotechnol. 11, 693–699. 10.1038/nnano.2016.70 27183057

[B40] Wu L. Liu H. Li J. Wang S. Wang X. (2019). A multi-level memristor based on AL-doped HfO2 thin film. Nanoscale Res. Lett. 14, 1–7. 10.1186/s11671-019-3015-x 31139948 PMC6538729

[B41] Yang G. Lai C. S. W. Cichon J. Ma L. Li W. Gan W.-B. (2014). Sleep promotes branch-specific formation of dendritic spines after learning. Science 344, 1173–1178. 10.1126/science.1249098 24904169 PMC4447313

[B42] Yang G. Pan F. Gan W.-B. (2009). Stably maintained dendritic spines are associated with lifelong memories. Nature 462, 920–924. 10.1038/nature08577 19946265 PMC4724802

[B43] Zenke F. Poole B. Ganguli S. (2017). Improved multitask learning through synaptic intelligence. arXiv preprint arXiv: 1703.04200. 10.48550/arXiv.1703.04200 PMC6944509 31909397

[B44] Zhou L. Chakrabartty S. (2017). Self-powered timekeeping and synchronization using Fowler-Nordheim tunneling-based floating-gate integrators. IEEE Trans. Electron Devices. 64, 1–7. 10.1109/TED.2016.2645379

[B45] Zhou L. Kondapalli S. H. Aono K. Chakrabartty S. (2019). Desynchronization of self-powered fn tunneling timers for trust verification of IoT supply chain. IEEE Internet Things J. 6, 6537–6547. 10.1109/JIOT.2019.2907930

