# Supplementary material for: On-device synaptic memory consolidation using Fowler-Nordheim quantum-tunneling
Source: Front Neurosci. 2023 Jan 13;16:1050585. doi: 10.3389/fnins.2022.1050585 (PMC9880265; doi:10.3389/fnins.2022.1050585)
Supplement: Supplementary file 1 [file Data_Sheet_1.pdf]

Supplementary Material

1 SUPPLEMENTARY DATA

1.1 Equivalent Circuit Model of FN-Synapse

The equivalent circuit model of a single FN-synapse is shown in Fig. S1. The synaptic weight W_d is stored as a difference between the voltages (W^+ and W^-) on the floating-gates. The FN tunneling current is modeled using voltage dependent current sources $J(W^+)$, $J(W^-)$ that discharge the floating-gate capacitances C_{fg}. Both W_d and the common-mode voltage W_c are estimated by measuring W^+ and W^- using a capacitive divider formed by C_1 and C_2 and respective source-followers A. This configuration has been previously demonstrated to avoid read-disturbances when measuring the floating-gate voltages Mehta et al. (2020); Zhou and Chakrabartty (2017). External input v_{in} is differentially coupled to the FN-synapse through the capacitances C_c and C_{mod} is used to couple the signal $m(t) = \frac{dv_{mod}(t)}{dt}$ common to all synapses. $m(t)$ is used to adjust the plasticity of the entire synaptic array. The initial charge on the floating-gates are programmed using a combination of FN quantum-tunneling and hot-electron injection, details of which can be found in Mehta et al. (2020).

1.2 Modeling Results

1.2.1 Behavioral Model of the FN-Synapse

The fabricated prototype of the FN-synapse array comprises of 64 FN-synaptic elements. Thus, for large-scale memory consolidation experiments and for large-scale continual learning experiments, we require a behavioral model that can accurately capture the response of each FN-synapse in the array. In our previous works Zhou et al. (2019); Zhou and Chakrabartty (2017) we have validated that equation 25 in the main manuscript can accurately (accuracy greater than 99%) model the dynamic response of a single FN tunneling junction and a corresponding integrator. For this work we instantiated two tunneling junctions corresponding to the floating-gates W^+ and W^- and the model parameters k_0, k_1 and k_2 were estimated using measured results. A non-linear regression was specifically used to estimate k_1 and k_2 Mehta et al. (2020); Zhou and Chakrabartty (2017), whereas k_0 was determined from the voltage to which each of the floating-gates were initialized. To validate the behavioral model of the FN-synapse, we carried out a set of experiments and compared the outputs against the analytical results shown in equations $33, 36 - 38$ in the Methods Section of the main manuscript. Note that these analytical expressions were derived for a constant modulation input, therefore $V_{mod}(t)$ was kept constant at $0V$ in all the simulated experiments. Fig S2 and S3 summarizes all the results obtained from the behavioral model.

In the first experiment, we measured the weight evolution of an FN-synapse using the fabricated prototype for a series of potentiation/depression pulses. The same input was provided to the software model and the weight evolution was simulated. Fig S2 (a) shows that the stored weight of the software model accurately matches with that of the hardware FN-synapse with a small deviation as shown in Fig S2 (b). This verifies that both hardware FN-synapse and software model behaves similarly when subjected to same stimuli. Next, we ran a Monte-Carlo simulation where we updated a network of $N = 10000$ FN-synapses with random binary pattern. Each tunneling junction of FN-synapses were initialized at $W_{c0} = 4.5V$. The updates were provided as a differential input voltage pulses of magnitude $4V$ and duration $\Delta t = 100ms$ to each synapses. The experiment was repeated for 1000 Monte-Carlo simulations. Fig S3 (a), (b), and (c) shows the SNR, memory retrieval signal $S(n)$ and the noise $\nu(n)$ respectively obtained from the software model of FN-synapse network. In Fig S3 (a) we observe that the SNR from the software model matches accurately

with the analytical expression. Both $S(n)$ and $\nu(n)$ described in equation 4 in the main manuscript have two different regimes depending on the value of γ. When $n << \gamma$, $S(n)$ is approximately constant and $\nu(n)$ increases at a rate of $\sqrt{n}$. On the other hand, when $n >> \gamma$, $S(n)$ and $\nu(n)$ falls off at a rate of $\frac{1}{n}$ and $\frac{1}{\sqrt{n}}$ respectively. Fig S3 (b) and (c) shows that the response from the software model follows theses trends and captures both the regimes accurately. In the next set of numerical experiments, we verified whether the FN-synapse network shows similar trends as the analytic expression in response to changing the value of γ in equation 5 in the main manuscript. Note that the parameter γ is defined as

$$\gamma = \frac{k_0}{k_1 \Delta t} \tag{S1}$$

where $k_0 = exp(\frac{k_2}{W_{c0}})$. Therefore, γ for the same set of FN-synapses increases when Δt or W_{c0} decreases and vice versa. According to equation 4, the value of n at which the regimes in these responses changes also shifts. Moreover, the initial values for both $S(n)$ and $\nu(n)$ depends on the value of γ while SNR is agnostic to changes in γ. Fig S3 (d)-(i) show the FN-synapse responses in relation to changing the pulse width and the initialization condition for a network size of $N = 1000$. From the figures we can observe that the software model is in very good agreement with the analytic expressions. Finally, we verify the behavioral model in relation to change in the size N of the FN-synapse network. From the analytic expressions in equation 4 in the main manuscript, $SNR \propto \sqrt{N}$ and $\nu(n) \propto \frac{1}{\sqrt{N}}$ while $S(n)$ remains constant with respect to N. Fig S3 (j)-(l) shows that the FN-synapse network exhibits these attributes accurately. Note that the regime switching point in $S(n)$ and $\nu(n)$ remains constant, since γ does not depend on the size of the network.

1.3 Plasticity and Consolidation

The ability of a network to learn new tasks is contingent on the availability of adequate range of plasticity of the synapses so that the weights learned from previous tasks can adapt sufficiently to reflect the requirements for the new tasks. Traditional volatile memories have practically infinite range of plasticity and can therefore change the weights stored to any extent that is required. However, this feature might not be beneficial for continual learning where the network needs to learn new tasks without forgetting the previous ones. This *rigidity-plasticity* dilemma is at the core underpinning of memory consolidation where more frequently used synapses become more rigid in comparison to the less frequently used synapses. Thus, a balance between the range of plasticity required to learn successive tasks and the consolidation of the weights learned in the process is key to continual learning. In the case of FN-synapse based neural networks, the range of plasticity is determined by the initial tunneling region of the device. A high tunneling region, denoted by a larger value of W_{c0}, ensures that the synapses are plastic enough to learn several successive tasks and slowly become rigid over time. This is seen in the case of W_{c0} = 5V and W_{c0} = 4.5V, which exhibit significantly better overall average accuracy over five tasks as shown in SI Fig. S5 (a) as the weights stored in their synapses (shown in SI Fig. S5 (b) and S5 (c) respectively) slowly spread from a highly plastic to a rigid region over the course of the five tasks. In contrast, a relatively low initial tunneling region, such as in the case of W_{c0} = 4V, does not learn new tasks as well as the previous couple of cases as shown in SI Fig. S5 (a) since in this case the weights stored in the synapse are already relatively rigid at the point of initiation and barely undergo any change as illustrated in SI Fig. S5 (d). Therefore by choosing the initial plasticity level appropriately we can achieve an optimal balance between the range of plasticity and consolidation suitable for continual learning. It is worth mentioning here that while choosing an appropriate temporal profile of $m(t)$ can be used to re-adjust the plasticity of the synapses after each update, it does not however change the range of plasticity afforded to the network since that is determined by the initial W_{c0}.

1.4 Neural Network Architecture

The architecture of the 4-layer fully-connected MLP is shown in SI Fig. S6 (a). Comprising an input layer of 1024 neurons corresponding to images of 32x32 pixels, two hidden layers of 80 and 60 neurons each and an output layer of 2 neurons that differentiates between (0,1) in t_1, (2,3) in t_2, (4,5) in t_3, (6,7) in t_4 and (8,9) in t_5 the network was constructed in MATLAB and trained with SGD and ADAM with learning rate of 0.001 for 4 epochs with a mini-batch size of 128. For comparisons with EWC and Online EWC, the network was replicated in python and trained with exactly the same parameters.

The evolution of the plasticity/usage of weights of the different layers of the FN-synapse based neural network are shown in SI Fig. S6 (b)-(d). Given the relatively large number of weights between layer 1-2 and layer 2-3, the amount of change in plasticity that they undergo (as shown in Fig S6 (b) and S6(c) respectively) is much lesser in comparison with those between layer 3-4 (as shown in Fig S6 (d)) as the presence of much fewer weights ensures that they are modified considerably frequently due to lack of any redundancy.

Fig. 6 of the main manuscript and SI Fig. S7 already depicts the advantages of the FN-synapse based neural networks using either SGD or ADAM as the optimizer when employed within the aforementioned architecture. In addition, if the size of the neural network is increased by increasing the number of neurons in the hidden layers from 80/60 in layer 2/3 to 400/400, it can be observed from SI Fig. S8 (a)-(b) that the average overall accuracy of the FN-synapse based network still outperforms the ones without it as the memory element. Interestingly, the accuracy of the larger network with FN-synapse is slightly lower than that of the smaller network with FN-synapse for task 3 and beyond. This dip is actually an indication of higher plasticity, and therefore slower consolidation, of the larger network due to presence of many more synapses which are still highly plastic after several tasks, which makes FN-synapse based large neural networks equipped with the capability of learning more complicated tasks than split-MNIST and yet exhibit far better consolidation than conventional memory.

1.5 Effects of Mismatch

The FN-synapse comprises of two differential FN tunneling junctions and the operation of the synapse assumes that the junctions are well matched. This will ensure that the weights stored in the synapse are equally plastic/rigid, when increasing or decreasing the magnitude of the weight. A key requirement is that the tunneling rates of the two junctions corresponding to W^+ and W^- are synchronized with each other. Previously, we have shown in Mehta et al. (2022, 2020) that two such FN-dynamical systems can be synchronized to a very high degree of accuracy even in the presence of temperature variations or device mismatch.

On the other hand, mismatch in device characteristics across one or more FN synapses, specifically the parameters k_1 and k_2, must be taken into consideration. This is because a neural network could comprise of billions of synapses and mismatch in synaptic behavior could pose a problem. SI Fig. S9 (a) shows the effect of a 5% mismatch in device characteristics across synapses on the SNR of an FN-synapse network comprising of 10,000 synapses. In this experiment, the network was subjected to 10,000 randomized balanced updates, similar to the previous consolidation experiments. It can be observed that the network with mismatch shows a small degradation in SNR or memory retention compared to the one without any mismatch. However, the SNR still follows the power-law curve. On the contrary a mismatch of 5% does not lead to any deterioration whatsoever of the average overall accuracy of the network when trained with SGD over the split-MNIST dataset with the incremental domain learning tasks as depicted in Fig S9 (b). This shows the robustness of the FN-synapse based network and the ability of learning to compensate for device mismatch.

1.6 Detailed Derivations

In this section, few additional steps were added to the modelling derivations in the main manuscript for the reader's interest. However, for the sake of brevity and to avoid repetition, unnecessary descriptions have been avoided.

1.6.1 Weight Update For Differential Synaptic Model

The state equations of two dynamical systems (corresponding to state variables W^+ and W^- with $J(.)$ defining their rate of change), when subjected to differential input $\pm\frac{1}{2}X(t)$ and common-mode modulation input $m(t)$ is given by:

$$\frac{dW^+}{dt} = -J(W^+) + \frac{1}{2}X(t) + \frac{1}{2}m(t) \tag{S2}$$

$$\frac{dW^-}{dt} = -J(W^-) - \frac{1}{2}X(t) + \frac{1}{2}m(t) \tag{S3}$$

Since, $W_d = \frac{W^+ - W^-}{2}$ and $W_c = \frac{W^+ + W^-}{2}$, S2 and S3 can be written as:

$$\frac{d(W_c + W_d)}{dt} = -J(W_c + W_d) + \frac{1}{2}X(t) + \frac{1}{2}m(t) \tag{S4}$$

$$\frac{d(W_c - W_d)}{dt} = -J(W_c - W_d) - \frac{1}{2}X(t) + \frac{1}{2}m(t) \tag{S5}$$

Then, by adding and subtracting S4 and S5, the following is obtained:

$$\frac{dW_c}{dt} = -\left(\frac{J(W_c + W_d) + J(W_c - W_d)}{2}\right) + m(t) \tag{S6}$$

$$\frac{dW_d}{dt} = -\left(\frac{J(W_c + W_d) - J(W_c - W_d)}{2}\right) + X(t) \tag{S7}$$

Upon applying Taylor series expansion on S6 and S7, with the assumption that $W_c >> W_d$, we get:

$$\frac{dW_c}{dt} = -J\left(W_c\right) + m(t) \tag{S8}$$

$$\frac{dW_d}{dt} = -J'\left(W_c\right) W_d + X(t) \tag{S9}$$

Therefore to obtain an expression of weight update ($\frac{dW_d}{dt}$) with respect to the common-mode usage (W_c), we need to obtain an expression for $J'(W_c)$. Thus, by differentiating S8 w.r.t t, we obtain:

$$\frac{d^2W_c}{dt^2} = -J'\left(W_c\right)\frac{dW_c}{dt} + m'(t) \tag{S10}$$

$$J'\left(W_c\right) = -\frac{\left(\frac{d^2W_c}{dt^2} - m'(t)\right)}{\frac{dW_c}{dt}} \tag{S11}$$

Inserting S11 into S9, we get:

$$\frac{dW_d}{dt} = -\left[\frac{\frac{d^2W_c}{dt^2} - m'(t)}{\frac{dW_c}{dt}}\right] W_d + X(t) \tag{S12}$$

Now, for the trivial case where $m(t) = c$, where c is an arbitrary constant, $m'(t) = 0$ and thus S12 becomes:

$$\frac{dW_d}{dt} = -\left[\frac{d^2W_c}{dt^2}\left(\frac{dW_c}{dt}\right)^{-1}\right] W_d + X(t) \tag{S13}$$

1.6.2 Optimal Usage Profile

The decay rate $(r(t))$ obtained from the weight update rule in S13 is given by:

$$r(t) = -\left[\frac{d^2W_c}{dt^2}\left(\frac{dW_c}{dt}\right)^{-1}\right] \tag{S14}$$

To avoid catastrophic forgetting, the decay rate associated with the EWC model's weight update rule, for the case of balanced inputs, is $r(t) = O\left(\frac{1}{t}\right)$. Therefore, by choosing $W_c = \frac{1}{f(\log t)}$, where $f(.) \geq 0$ is a monotonic function we obtain

$$r(t) = \frac{1}{t}\left(1 + \frac{2f'(\log t)}{\log t} - \frac{f''(\log t)}{f'(\log t)}\right) \tag{S15}$$

which is of the order $O\left(\frac{1}{t}\right)$. The simplest form of $f(.)$ such that W_c satisfies both monotonicity and the order of decay, is given by:

$$W_c = \frac{\beta}{\log(t)} \tag{S16}$$

where β is an arbitrary constant. Consequently, to obtain the non-linear function $J(.)$ which enforces the above constraint, we substitute S16 into S8 to get:

$$\frac{d\left(\frac{\beta}{\log(t)}\right)}{dt} = -J(W_c) + m(t) \tag{S17}$$

$$\frac{-\beta}{t(log(t))^2} = -J(W_c) + m(t) \tag{S18}$$

For the case of $m(t) = 0$, S18 becomes:

$$J(W_c) = \frac{\beta}{t(log(t))^2} \tag{S19}$$

Now, from S16, we can obtain an expression for $log(t)$ as

$$log(t) = \frac{\beta}{W_c} \tag{S20}$$

and an expression for t as follows:

$$exp(log(t)) = exp\left(\frac{\beta}{W_c}\right) \tag{S21}$$

$$t = exp\left(\frac{\beta}{W_c}\right) \tag{S22}$$

Then, by substituting S20 and S22 in S19, we obtain:

$$J\left(W_c\right) = \frac{1}{\beta} W_c^2 \exp\left(-\frac{\beta}{W_c}\right). \tag{S23}$$

1.6.3 Signal-to-noise Ratio Estimation for Random Pattern Experiment

The weight update equation for an FN-synapse (similar to S13) is given by:

$$C_T \frac{dW_d}{dt} = -\left[\frac{d^2 W_c}{dt^2}\left(\frac{dW_c}{dt}\right)^{-1}\right] W_d + C_c \frac{dv_{in}}{dt} \tag{S24}$$

where $C_T = f(C_1, C_2, C_{fg})$ is the cumulative capacitance and C_c is the coupling capacitance of the FN-synapse equivalent circuit as shown in Fig. S1. Since, the physics of FN-tunneling leads to a common-mode voltage W_c profile such that

$$W_c(t) = \frac{k_2}{\log(k_1 t + k_0)} \tag{S25}$$

where $k_0 = \exp\left(\frac{k_2}{W_{c0}}\right)$ and W_{c0} refers to the initial voltage at the floating-gate, by substituting S25 in S24, we get:

$$C_T \frac{dW_d}{dt} = -\left[\frac{\left(\frac{k_1^2 k_2}{(k_1 t + k_0)^2 log^2(k_1 t + k_0)}\right)}{\left(\frac{k_1 k_2}{(k_1 t + k_0) log^2(k_1 t + k_0)}\right)}\left(1 + \frac{2}{log(k_1 t + k_0)}\right)\right] W_d + C_c \frac{dv_{in}}{dt} \tag{S26}$$

$$C_T \frac{dW_d}{dt} = -\left[\left(\frac{k_1}{(k_1 t + k_0)}\right)\left(1 + \frac{2}{log(k_1 t + k_0)}\right)\right] W_d + C_c \frac{dv_{in}}{dt} \tag{S27}$$

In the scenario where $C_T = C_c$, we get:

$$\frac{dW_d}{dt} = -\left[\left(\frac{k_1}{(k_1 t + k_0)}\right)\left(1 + \frac{2}{log(k_1 t + k_0)}\right)\right] W_d + \frac{dv_{in}}{dt} \tag{S28}$$

Then, we can formulate a discrete-time weight update as:

$$\begin{aligned}\frac{\triangle W_d(n)}{\triangle t} &= -k_1\left(1+\frac{2}{\log\left(k_1\triangle tn+k_0\right)}\right)\left(\frac{1}{k_1\triangle tn+k_0}\right)W_d(n-1)\\ &+\frac{\triangle v_{in}(n)}{\triangle t}\end{aligned} \tag{S29}$$

$$\begin{aligned}W_d(n) &= \left[1-\left(1+\frac{2}{\log\left(k_1\triangle tn+k_0\right)}\right)\left(\frac{1}{n+\frac{k_0}{k_1\triangle t}}\right)\right]W_d(n-1)\\ &+\left(v_{in}(n)-v_{in}(n-1)\right)\end{aligned} \tag{S30}$$

where n represents the number of patterns observed and Δt is the duration of the input pulse. Let us denote the weight decay term as

$$\alpha(n) = \left[1-\left(1+\frac{2}{\log\left(k_1\triangle tn+k_0\right)}\right)\left(\frac{1}{n+\frac{k_0}{k_1\triangle t}}\right)\right] \tag{S31}$$

Thus, we obtain the weight update equation with respect to number of patterns observed as

$$W_d(n) = \alpha(n)W_d(n-1)+\left(v_{in}(n)-v_{in}(n-1)\right) \tag{S32}$$

Then the equation can be unfolded as follows:

$$W_d(n) = \alpha(n)W_d(n-1)+\left(v_{in}(n)-v_{in}(n-1)\right) \tag{S33}$$

$$W_d(n-1) = \alpha(n-1)W_d(n-2)+\left(v_{in}(n-1)-v_{in}(n-2)\right) \tag{S34}$$

and so on, till ...

$$W_d(2) = \alpha(2)W_d(1)+\left(v_{in}(2)-v_{in}(1)\right) \tag{S35}$$

$$W_d(1) = \alpha(1)W_d(0)+\left(v_{in}(1)-v_{in}(0)\right) \tag{S36}$$

Assuming the initial condition that $W_d(0)=0$ and $x(0)=0$, if we multiply each $W_d(i)$ with the product of all $\alpha(i)$s succeeding it and sum them up, we get:

$$\begin{aligned}W_d(n) &= \left(v_{in}(n)-v_{in}(n-1)\right)+\alpha(n)\left(v_{in}(n-1)-v_{in}(n-2)\right)\\ &+\alpha(n)\alpha(n-1)\left(v_{in}(n-2)-v_{in}(n-3)\right)+...\\ &+\alpha(n)\alpha(n-1)...\alpha(4)\alpha(3)\left(v_{in}(2)-v_{in}(1)\right)\\ &+\alpha(n)\alpha(n-1)...\alpha(3)\alpha(2)v_{in}(1)\end{aligned} \tag{S37}$$

This can be generalized as

$$\begin{aligned}W_d(n) &= \{v_{in}(n)+(\alpha(n)-1)v_{in}(n-1)\\ &+(\alpha(n-1)-1)\alpha(n)v_{in}(n-2)\\ &+...\\ &+\alpha(n)\alpha(n-1)...\alpha(3)(\alpha(2)-1)v_{in}(1)\}\end{aligned} \tag{S38}$$

$$W_d(n) = \sum_{i=1}^{n-2}\left\{(\alpha(i+1)-1)\left(\prod_{j=i+2}^{n}\alpha(j)\right)v_{in}(i)\right\} + (\alpha(n)-1)v_{in}(n-1) + v_{in}(n) \tag{S39}$$

Therefore, each weight $W_d(n)$ at time instance n can be represented as a summation of the product of synaptic modifications or patterns $v_{in}(n-1), v_{in}(n-2)...v_{in}(1)$ and cumulative decay rate $r_c(n, n-1), r_c(n, n-2), ...r_c(n, 1)$ for instances preceeding n as:

$$W_d(n) = \sum_{i=1}^{n-1} v_{in}(i) r_c(n,i) + v_{in}(n) \tag{S40}$$

where

$$r_c(n,i) = (\alpha(i+1)-1)\left(\prod_{j=i+2, j\leq n}^{n}\alpha(j)\right) \tag{S41}$$

Then, for a network of N synapses, each indexed as $W_d(a, n)$ (where $a = 1, ..., N$), with the input applied to the ath synapse after n patterns represented by $v_{in}(a, n)$, the signal strength for the pth update (where $p < n$) tracked after n patterns is given by:

$$S(n,p) = \frac{1}{N}\left\langle \sum_{a=1}^{N} W_d(a,n) v_{in}(a,p)\right\rangle \tag{S42}$$

where angle brackets denote averaging over the ensemble of all of the random uncorrelated patterns seen by the network. Since, the signal corresponding to a certain update is essentially determined by the overlap of the associated history of synaptic modifications with the present synaptic weights, by substituting S40 into S42, we get the signal strength of the pth update as:

$$S(n,p) = \frac{1}{N}\left\langle \sum_{a=1}^{N} W_d(a,n) v_{in}(a,p)\right\rangle = r_c(n,p) = (\alpha(p+1)-1)\prod_{j=p+2}^{n}\alpha(j) \tag{S43}$$

Given that in S31, $k_0 = \mathcal{O}(10^{19})$ and $k_1 = \mathcal{O}(10^{16})$, the term $\left(1 + \frac{2}{\ln(k_1 \triangle tn + k_0)}\right) \approx 1$, the above equation can be simplified as follows:

$$\begin{aligned} S(n,p) &= \frac{-1}{p+1+\gamma}\left(1-\frac{1}{p+2+\gamma}\right)\left(1-\frac{1}{p+3+\gamma}\right) \\ &...\left(1-\frac{1}{n-1+\gamma}\right)\left(1-\frac{1}{n+\gamma}\right) \\ S(n,p) &= \frac{-1}{n+\gamma} \end{aligned} \tag{S44}$$

where $\gamma = \frac{k_0}{k_1 \triangle t}$.This leads to the following expression for signal power:

$$S^2(n,p) = \frac{1}{(n+\gamma)^2} \tag{S45}$$

By assuming that the weight $W_d(n)$ is uncorrelated from the input pattern $v_{in}(n)$ and that the inputs $v_{in}(1), v_{in}(2)...v_{in}(n)$ are all uncorrelated from each other, we can obtain the noise power associated with the retrieved signal (which is essentially the variance of the retrieved signal). It is measured as the summation of the power of all signals tracked at n except for the retrieval signal of the pth pattern and is expressed as:

$$\nu^2(n,p) = \frac{1}{N} \sum_{i=1, i \neq p}^{n} S^2(n,i) \tag{S46}$$

By incorporating the retrieval signal into the summation in S46 we can obtain a more tractable analytical expression for noise power despite the marginal error it introduces. The resulting expression is given by

$$\nu^2(n,p) = \frac{1}{N} \sum_{i=1}^{n} S^2(n,i) = \frac{n}{N(n+\gamma)^2} \tag{S47}$$

Based on the value of n in comparison to γ, we obtain two trends for the noise profile. When $\gamma >> n$,

$$\nu(n,p) = \frac{1}{\sqrt{N}} \left(\frac{\sqrt{n}}{\gamma} \right) \tag{S48}$$

which implies that noise increases with increase in updates initially. On the other hand, when $\gamma << n$,

$$\nu(n,p) = \frac{\sqrt{n}}{\sqrt{N} n} = \frac{1}{\sqrt{N}} \left(\frac{1}{\sqrt{n}} \right) \tag{S49}$$

which implies that noise falls with increase in updates in the later stages. The signal-to-noise ratio (SNR) of a network of size N can then be obtained as:

$$SNR(n,p) = \sqrt{\frac{S^2(n,p)}{\nu^2(n,p)}} = \sqrt{\frac{N}{n}} \tag{S50}$$

REFERENCES

Mehta, D., Aono, K., and Chakrabartty, S. (2020). A self-powered analog sensor-data-logging device based on fowler-nordheim dynamical systems. *Nature Communications* 11. doi:10.1038/s41467-020-19292-w

Mehta, D., Rahman, M., Aono, K., and Chakrabartty, S. (2022). An adaptive synaptic array using fowler–nordheim dynamic analog memory. *Nature Communications* 13, 1–11

Zhou, L. and Chakrabartty, S. (2017). Self-powered timekeeping and synchronization using fowler-nordheim tunneling-based floating-gate integrators. *IEEE Transactions on Electron Devices* PP, 1–7. doi:10.1109/TED.2016.2645379

Zhou, L., Kondapalli, S. H., Aono, K., and Chakrabartty, S. (2019). Desynchronization of self-powered fn tunneling timers for trust verification of iot supply chain. *IEEE Internet of Things Journal* 6, 6537–6547

2 SUPPLEMENTARY FIGURES

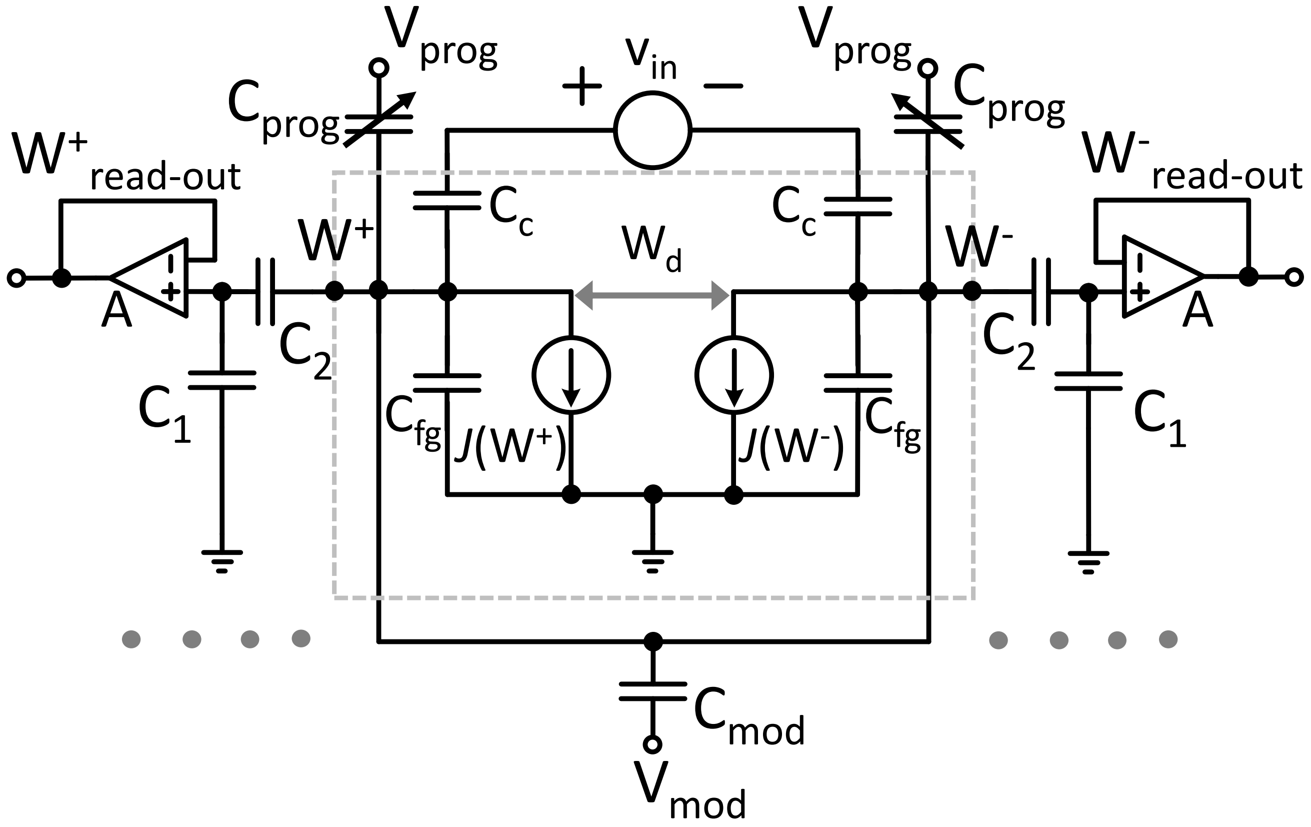

Figure S1. Equivalent circuit model of an FN-synapse

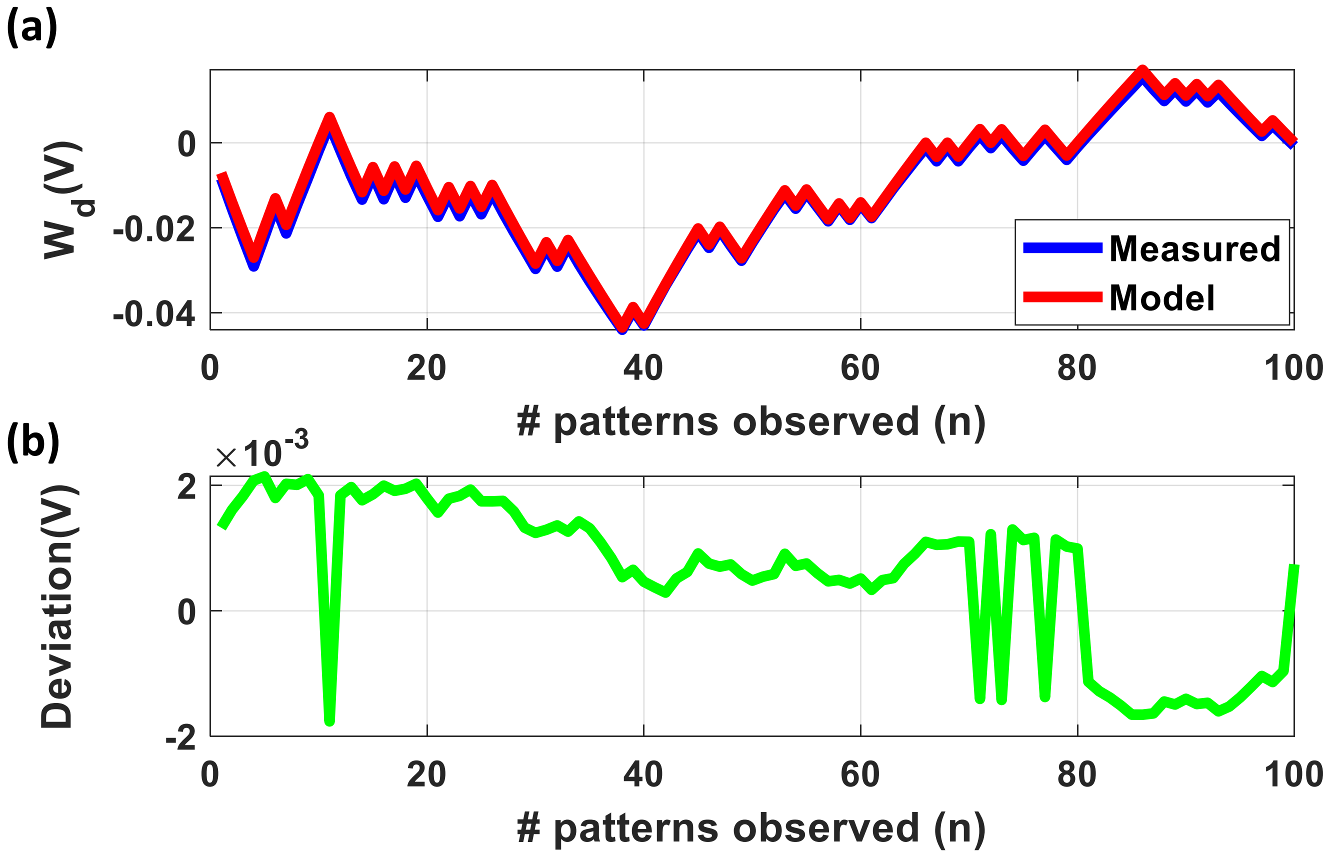

Figure S2. (a) Comparison of weight (W_d) stored in the FN-synapse and its software model of equivalent plasticity and initial conditions when exposed to the same input pattern and (b) the corresponding deviation.

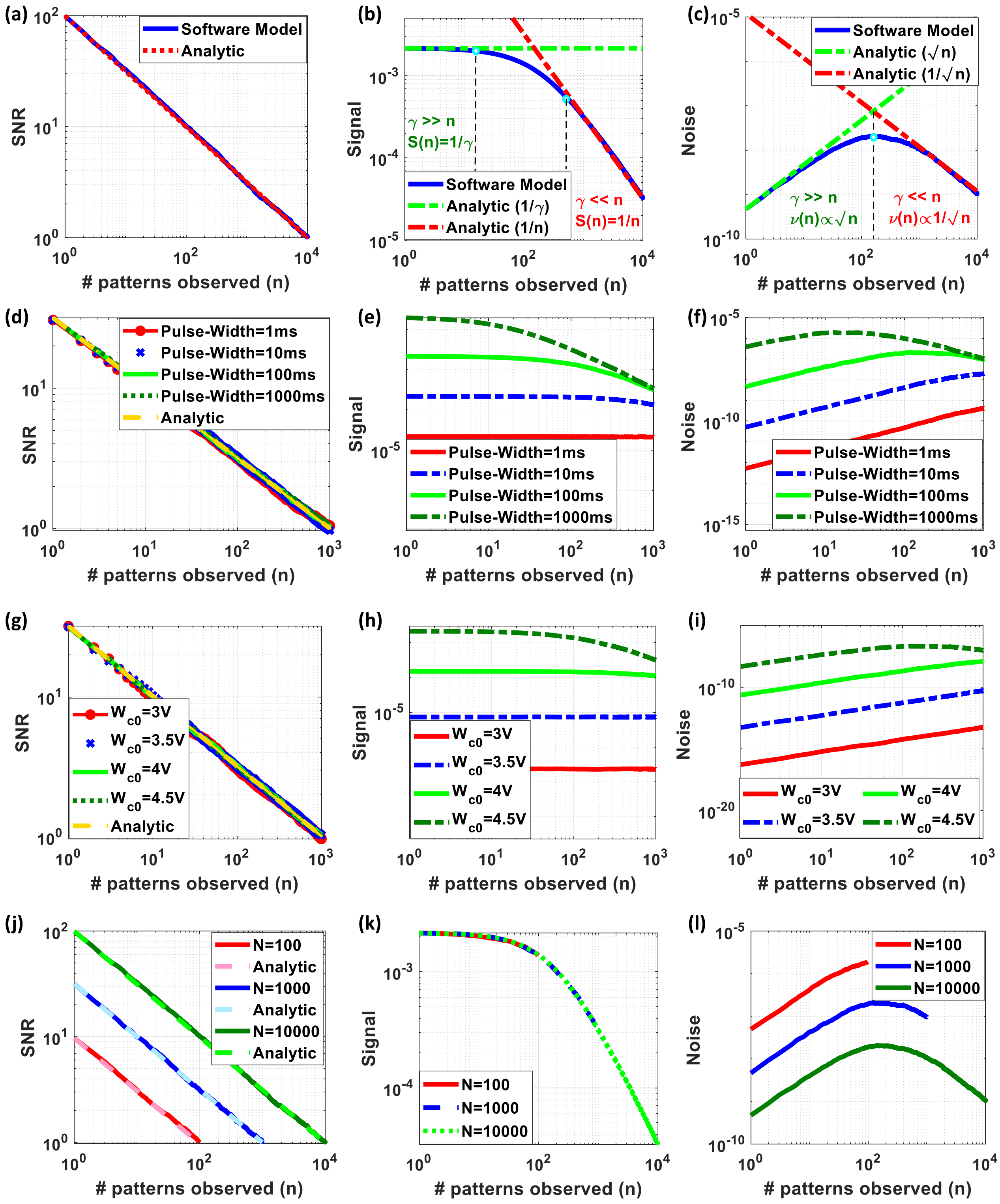

Figure S3. Comparison between the behavioral model and the analytical model of the FN-synapse in terms of (a) SNR, (b) signal and (c) noise. The effect on the SNR, signal and noise of the software model when (d)-(f) the pulse-width of the input pulse is varied and when (g)-(i) the magnitude of the input pulse is varied. (j)-(l) The impact of change in network size on SNR, signal and noise .

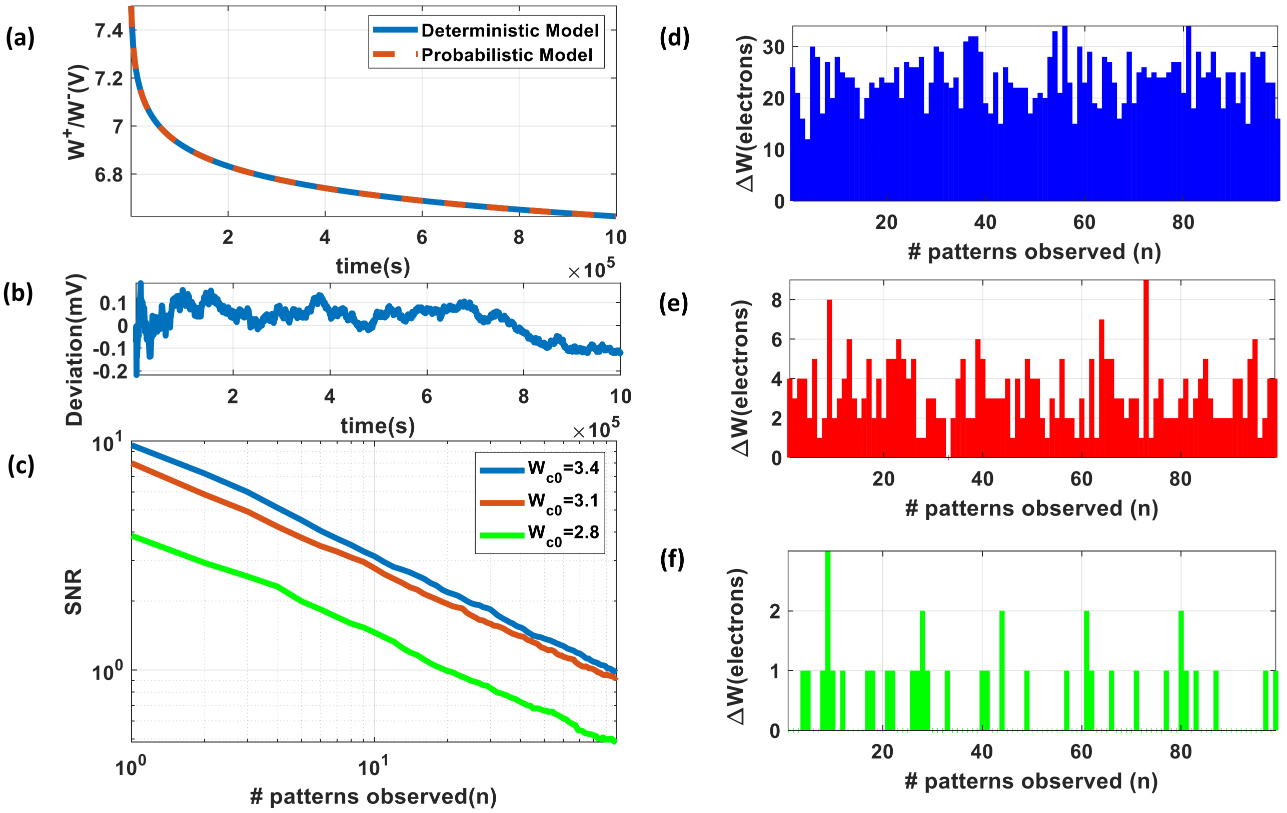

Figure S4. (a) Comparison between the output of the probabilistic FN-synapse model and the deterministic behavioral model and the (b) corresponding deviation. (c) The SNR of the network for different tunneling regions for W_{c0} = 3.4V, 3.1V and 2.8V and (d)-(f) their corresponding update size in terms of no. of electrons per update.

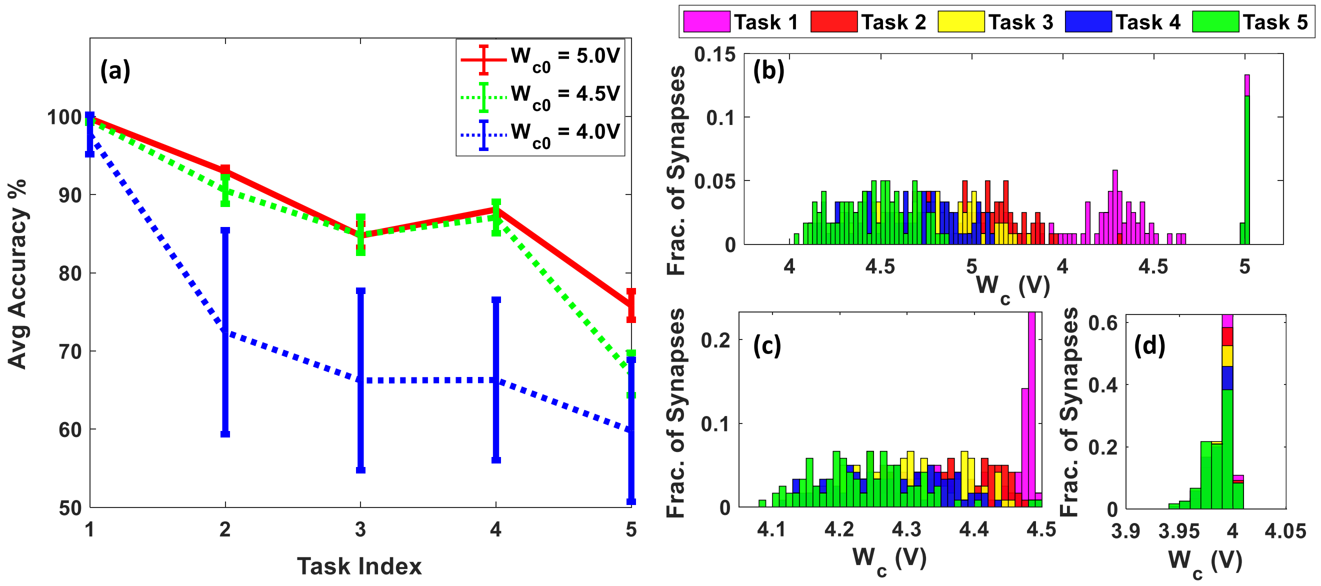

Figure S5. Effect of initial plasticity (W_{c0}) of FN-synapse on (a) overall average accuracy of the split-MNIST incremental domain learning tasks as a result of the degree of change in plasticity of their corresponding weights for (b) W_{c0} = 5.0V, (c) W_{c0} = 4.5V and (d) W_{c0} = 4.0V.

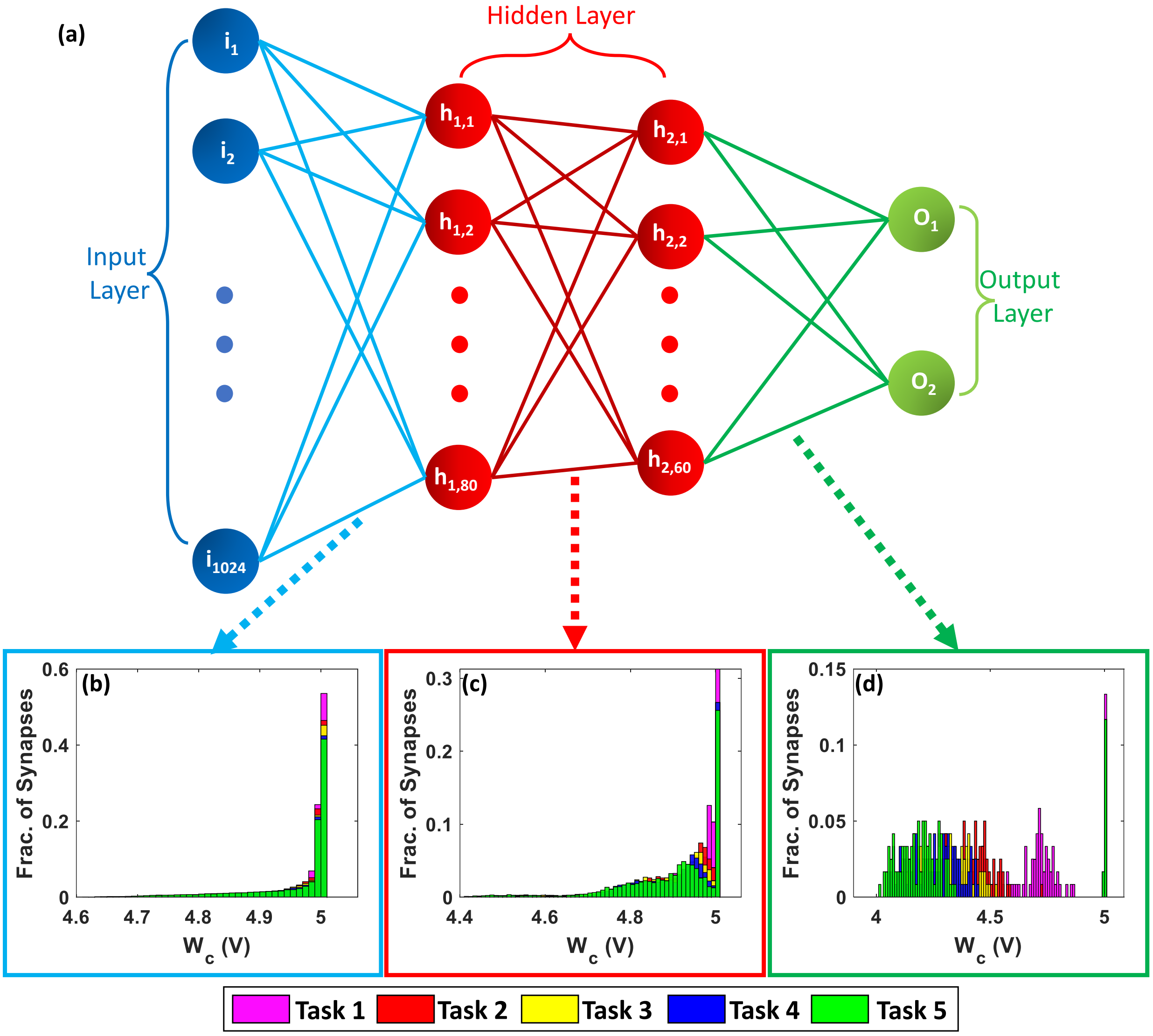

Figure S6. (a) The architecture of the neural network used in the report and the evolution of corresponding weights in between (b) layer 1 and 2, (c) layer 2 and 3, and (d) layer 3 and 4 over five successive tasks.

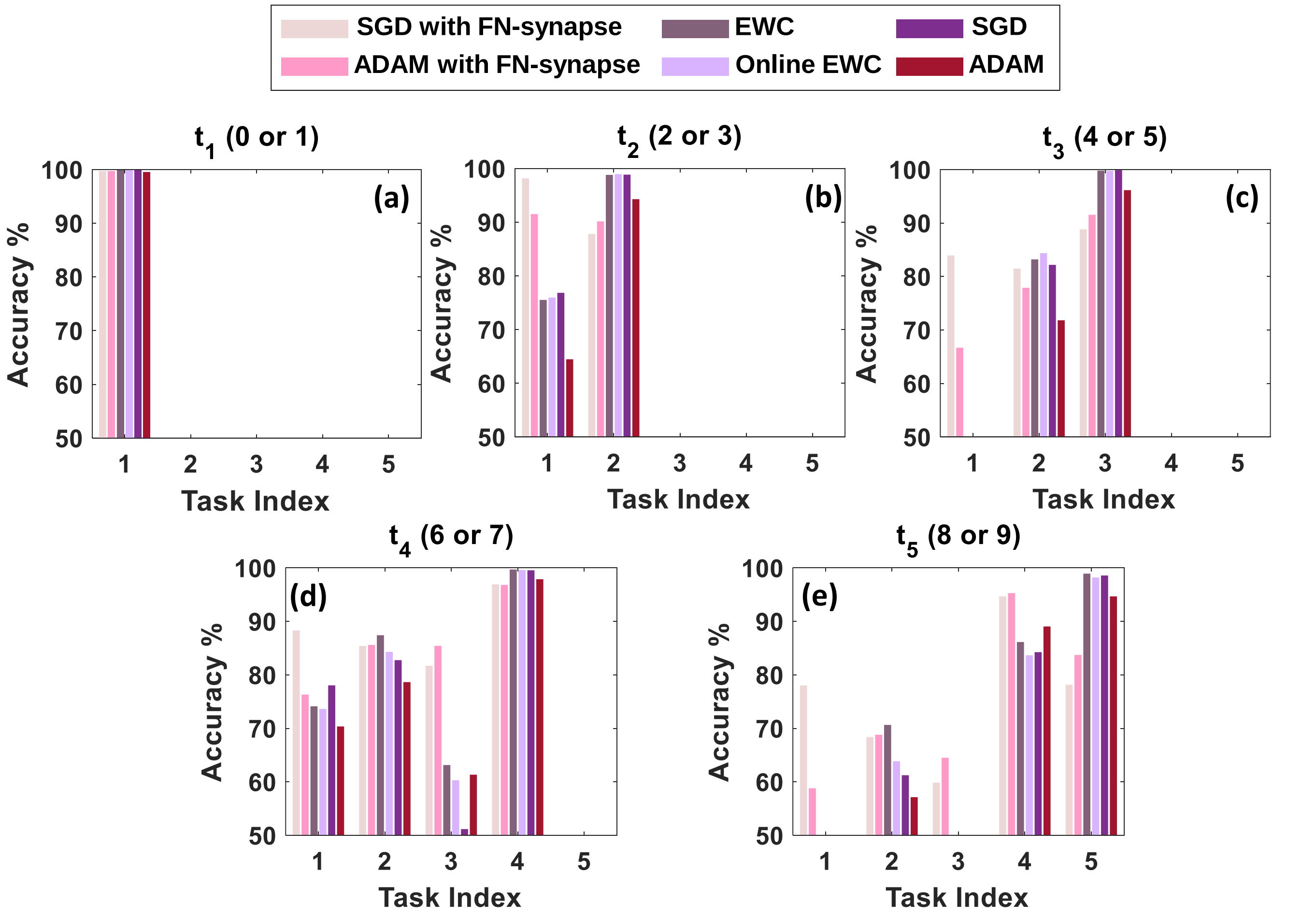

Figure S7. (a)-(e) Task-wise accuracy comparison of SGD and ADAM with FN-synapse, ADAM with EWC and Online EWC, SGD and ADAM with conventional memory.

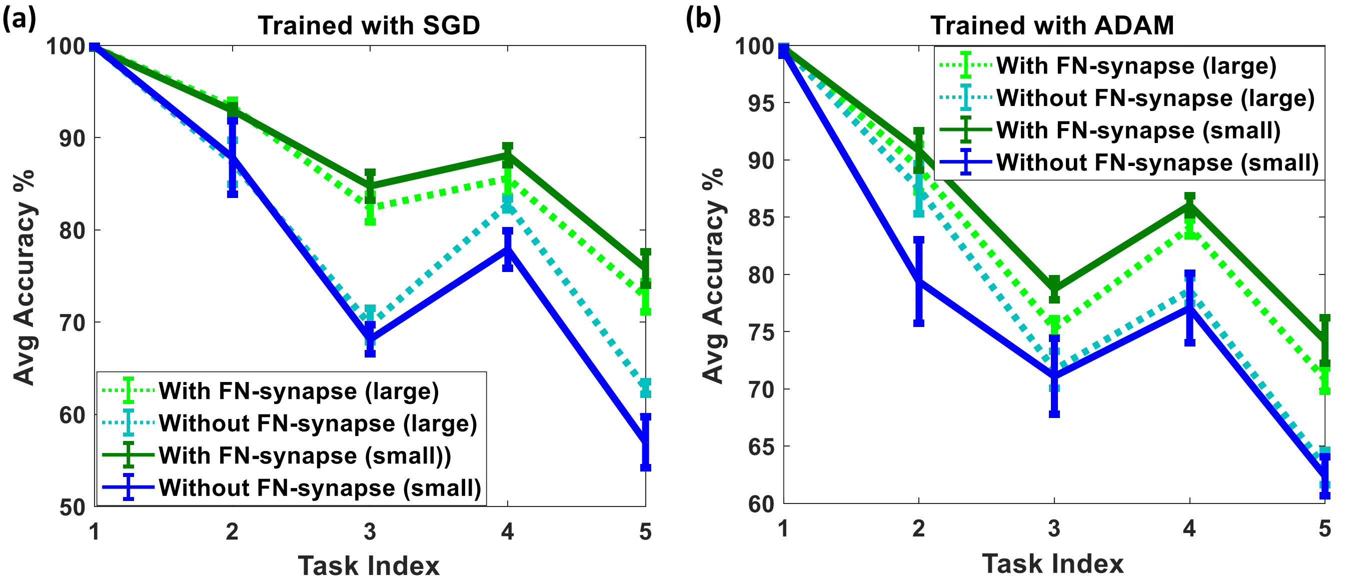

Figure S8. Effect of network size on overall average accuracy when trained with (a) SGD and (b) ADAM.

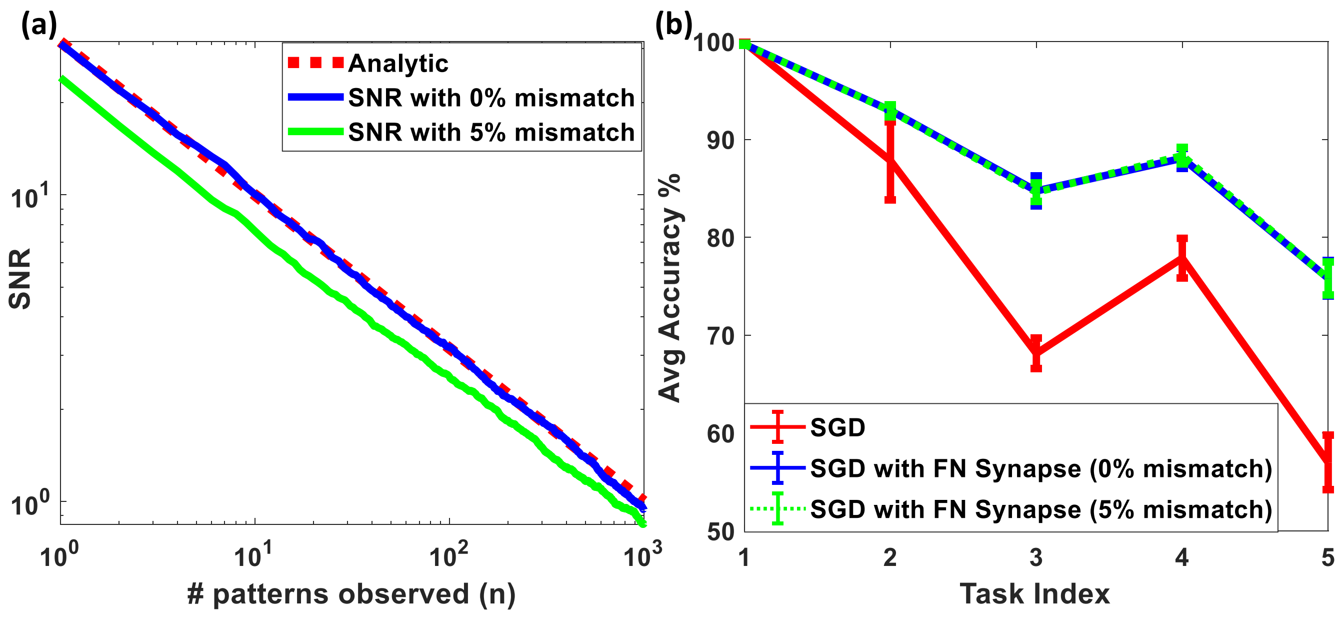

Figure S9. Effect of mismatch in device characteristics across FN synapses on (a) memory retention and (b) learning ability on the split-MNIST based incremental domain learning tasks.

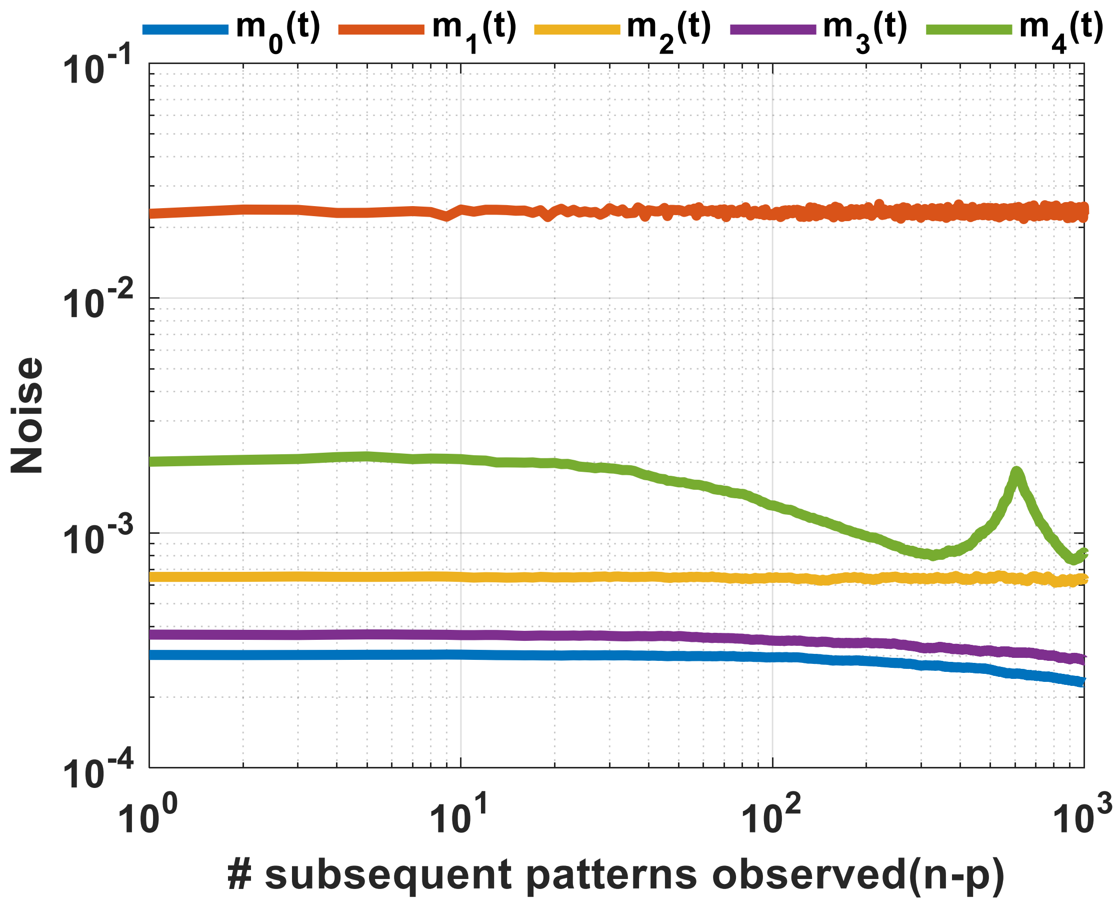

Figure S10. Comparison of noise of FN-synapse networks composed of 1000 synapses following different synaptic models when exposed to 2000 patterns.

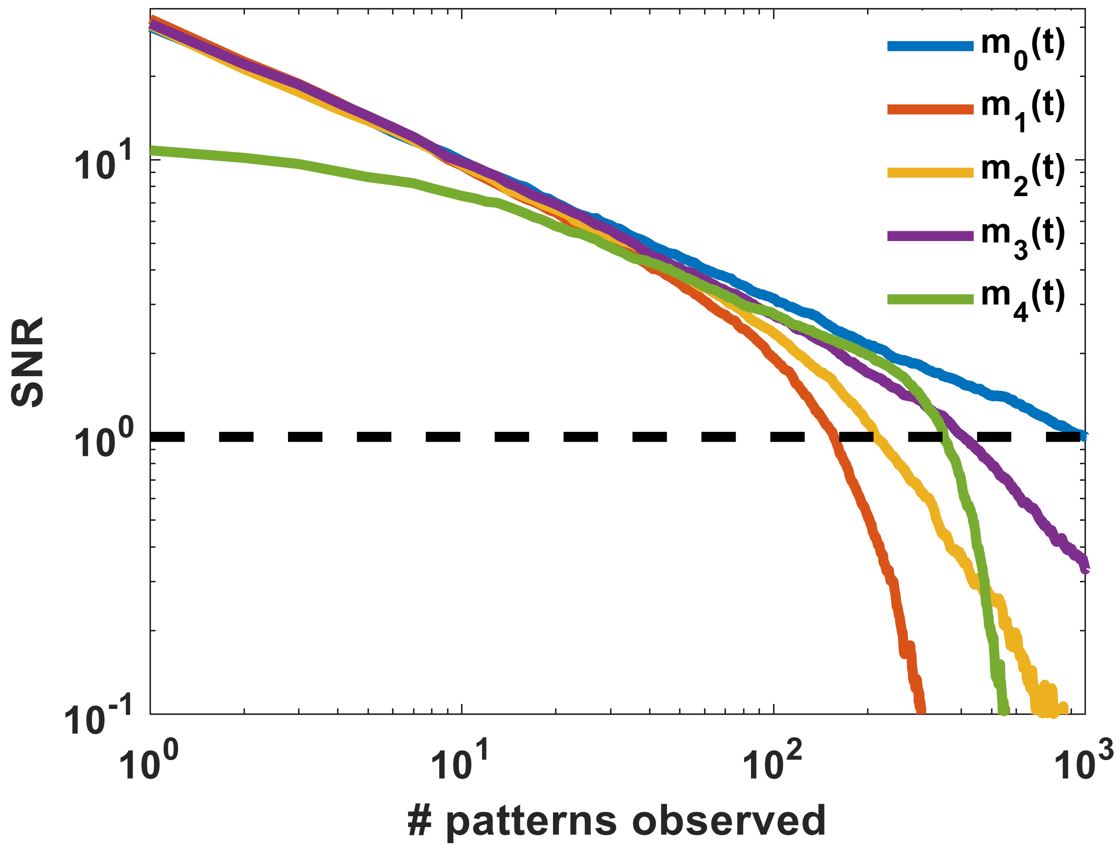

Figure S11. Comparison of SNR of an empty network of 1000 synapses with different modulation profiles $m(t)$ when exposed to 2000 patterns.

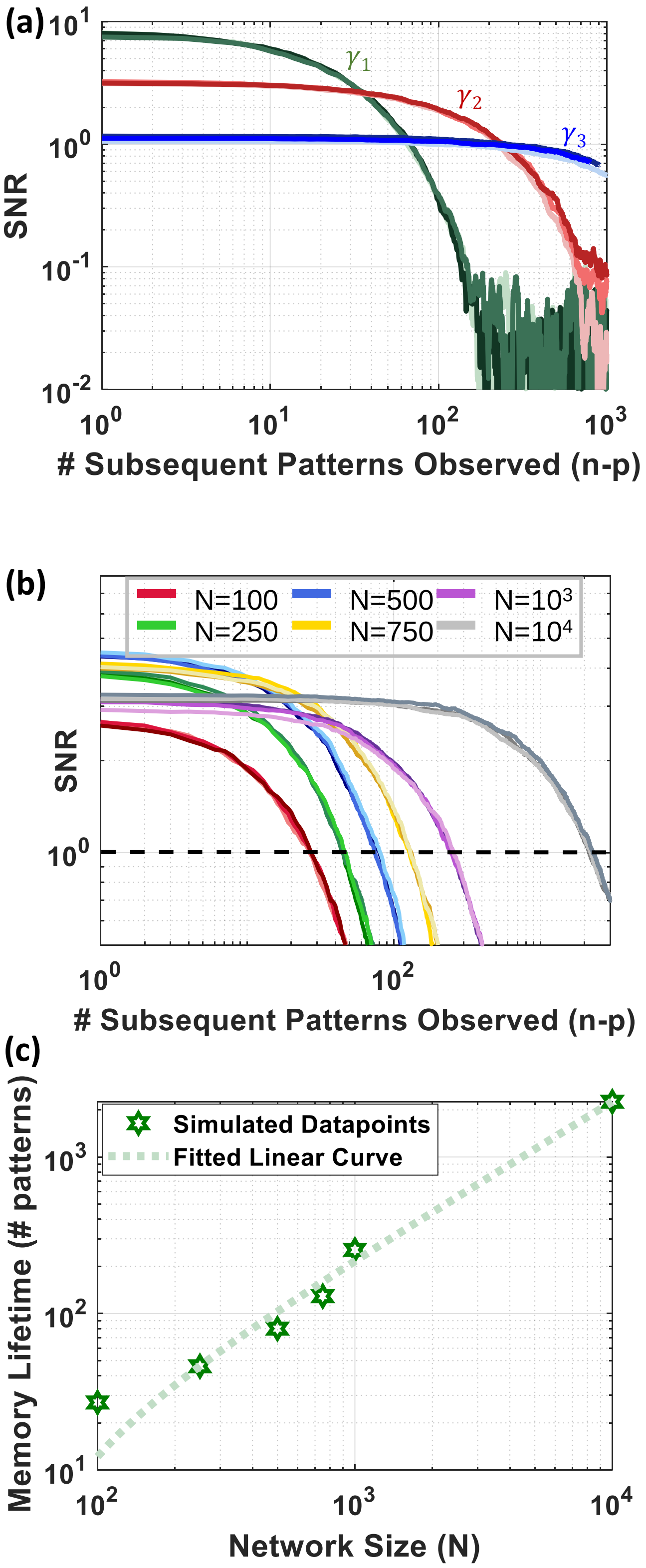

Figure S12. (a) The SNR in the steady state for an FN-synapse network of size $N = 1000$ with different magnitude of γ where $\gamma_3 > \gamma_2 > \gamma_1$ under modulation profile of $m_2(t)$. The magnitude of γ was varied by using three different input modulation pulse width Δt.(b) Tracking the steady-state SNR of various updates (p) for FN-synapse networks of different sizes (N) with modulation profile $m_2(t)$ when exposed to subsequent updates and (c) their corresponding memory lifetime which scales linearly according to $y = mx + c$, where $m = 0.2264$ and $c = -10.46$.
